# Expression of LGR5, FZD7, TROY, and MIST1 in Perioperatively Treated Gastric Carcinomas and Correlation with Therapy Response

**DOI:** 10.1155/2019/8154926

**Published:** 2019-11-19

**Authors:** Antonia Freiin Grote, Christine Halske, Hans-Michael Behrens, Sandra Krüger, Franziska Wilhelm, Jan-Hendrik Egberts, Christoph Röcken

**Affiliations:** ^1^Department of Pathology, Christian-Albrechts-University, Kiel, Germany; ^2^Department of General Surgery, Visceral, Thoracic, Transplantation and Pediatric Surgery, University Hospital Schleswig-Holstein (UKSH), Kiel, Germany

## Abstract

The cancer stem cell model is considered as a putative cause of resistance to chemotherapy and disease recurrence in malignant tumors. In this study, we tested the hypothesis that the response to neoadjuvant/perioperative chemotherapy correlates with the expression of four different putative cancer stem cell markers of gastric cancer (GC), i.e., LGR5, FZD7, TROY, and MIST1. The expression of LGR5, FZD7, TROY, and MIST1 was assessed by immunohistochemistry in 119 perioperatively treated GCs including pretherapeutic biopsies, resected primary GCs, and corresponding nodal and distant metastases. All four markers were detected in our cohort with variable prevalence and histoanatomical distributions. Few tumor cells expressed TROY. LGR5, FZD7, and MIST1 were coexpressed in 41.2% and completely absent in 6.2%. The prevalence of LGR5- and FZD7-positive GCs was higher and of TROY-positive GCs lower in perioperatively treated GCs compared with treatment-naïve tumors. LGR5, FZD7, and MIST1 in the primary tumors correlated significantly with their expression in the corresponding lymph node metastasis. An increased expression of LGR5 in primary GC correlated significantly with tumor regression. The expression of MIST1 in lymph node metastases correlated significantly with the number of lymph node metastases as well as overall and tumor-specific survival. FZD7 did not correlate with any clinicopathological patient characteristic. Our study on clinical patient samples shows that GCs may coexpress independently different stem cell markers; that neoadjuvant/perioperative treatment of GC significantly impacts on the expression of stem cell markers, which cannot be predicted by the analysis of pretherapeutic biopsies; and that their expression and tumor biological effect are heterogeneous and have to be viewed as a function of histoanatomical distribution.

## 1. Introduction

Gastric cancer (GC) is a leading cause of cancer death worldwide [1]. In Western countries, GC is often diagnosed at an advanced stage, leading to an overall poor prognosis [2]. Several studies have shown that patients with limited metastatic disease benefit from neoadjuvant/perioperative chemotherapy [3–5]. However, therapy response is unpredictable, and complete (Becker regression grade 1a) or subtotal (Becker regression grade 1b) response is achieved only in less than 30% of the patients [6].

The tumor stem cell hypothesis assumes that chemotherapy leads to a selective survival of resistant cancer stem cells (CSC), which are protected by different mechanisms from the effects of chemotherapy [7]. CSCs are then able to initiate tumor regrowth leading to tumor recurrence [7]. The resistance of CSCs to conventional chemotherapeutic agents has been demonstrated in a large number of studies [8–10], raising hopes that CSCs may serve as predictive or prognostic markers of therapeutic efficacy. However, evidence in clinical samples is scarce often due to the lack of appropriate biomarkers to screen for CSCs in tissue samples. With regard to GC, four different molecules had been suggested to be CSC markers.

The G-protein-coupled receptor LGR5 (leucine-rich repeat-containing G-protein-coupled receptor 5) is a target gene of the WNT signaling pathway, which can lead to an amplification of the WNT/*β*-catenin signal via the binding of R-spondins [11, 12]. LGR5 is a marker of adult stem cells of the stomach, the hair follicles, the small intestine, and the colon [13–15]. Several studies have already shown an association between LGR5 expression in GC and increased tumor progression, metastasis, and worse prognosis [16, 17]. LGR5 has also been implicated in the chemotherapy resistance of various cancers, as well as in GC [18–21].

FZD7 (Frizzled-7) is another target gene of the WNT signaling pathway that can activate the WNT signaling pathway in the presence of the coreceptor LRP [22, 23]. It was shown that FZD7 is involved in the maintenance of stem cell activity in embryonic stem cells [24]. FZD7 is vital for tissue homeostasis in the gastric epithelium: deletion of FZD7 in the mouse model leads to a dramatic reduction of mucus-secreting cells. FZD7 may regulate Muc5a expression and thus the differentiation of mucus-secreting cells. Deletion of FZD7 also leads to gastric repopulation [25]. In GC, upregulation of FZD7 was detected and associated with tumor progression, metastasis, and poor prognosis [26–28]. Recently, it was shown that FZD7 is the predominant Wnt receptor responsible for transmitting Wnt signaling in gastric tumor cells and plays an essential role in tumorigenesis [29]. Li et al. described FZD7 as an important factor in the CSC activity of GC [27]. Apart from GC, dysregulation of FZD7 was also observed in, e.g., colon, hepatocellular, and breast cancers [30–32]. For hepatocellular carcinoma and squamous cell carcinoma of the esophagus, an association between FZD7 expression and lack of response has been shown [33, 34]. FZD7 also regulates the function of LGR5 stem cells in the intestine [35].

TROY (tumor necrosis factor receptor superfamily, member 19), also a target gene of the WNT signaling pathway, causes negative feedback and thus indirect inhibition of the signaling pathway [36, 37]. TROY is important in the development of hair follicles and embryonic skin [38, 39]. Furthermore, in the stomach, a group of TROY expressing chief and parietal cells are present at the gland base of the corpus, of which the TROY expressing chief cells had abilities of self-contained reserve stem cells [37, 40]. In addition, dysregulation of TROY was observed in malignant melanoma, glioblastoma, and GC [37, 41, 42]. There was a significantly more frequent expression of TROY in GC in well to moderately differentiated tumors, intestinal carcinomas, and tumors without lymph node metastases [37]. Moreover, patients with a lack of TROY expression had a worse prognosis [37].

MIST1 (muscle, intestine, and stomach expression 1/BHLHA15) is a transcription factor belonging to the family of bHLH proteins [43]. It is involved in the development of the exocrine pancreas, liver, and stomach [43–45]. In the adult murine stomach, Hayakawa et al. found a slowly dividing subpopulation of MIST1-positive isthmus cells of the corpus gland, which could represent the origin of all cell lines in the corpus epithelium by bidirectional migration [46]. However, differentiated zymogenic chief cells at the base of the corpus glands did not show any stem cell properties [46]. In addition, they showed that the MIST1-positive isthmus cells can be the starting point of intestinal and diffuse GC [46]. Moreover, in the isthmus area of the antrum, a group of long-lived MIST1-positive progenitor cells were found, which were largely independent of other stem cell populations and can serve as an origin of antral tumors [47].

In this study, we tested the hypothesis that the response to neoadjuvant/perioperative chemotherapy correlates with the expression of four different CSC markers of GC, i.e., LGR5, FZD7, TROY, and MIST1 by using clinical samples.

## 2. Materials and Methods

### 2.1. Ethics Statement

The study was approved by the local ethical review board (D 525/15). All patient data were pseudonymized.

### 2.2. Study Population

From the archive of the Institute of Pathology, University Hospital Kiel, we sought all patients who had undergone either total or partial gastrectomy for adenocarcinoma of the stomach or esophagogastric junction between 1998 and 2016. The following patient characteristics were retrieved: type of surgery, age at diagnosis, gender, tumor size, tumor localization, tumor type, depth of invasion, number of lymph nodes resected, and number of lymph nodes with metastases [48]. Patients were included if an adenocarcinoma of the stomach or esophagogastric junction was histologically confirmed and the patients had undergone neoadjuvant or perioperative chemotherapy. Exclusion criteria were defined as follows: (1) histology identified a tumor type other than adenocarcinoma and (2) patients had not received a perioperative or neoadjuvant chemo- or radiotherapy. Each resected specimen had undergone gross sectioning and histological examination by trained and board-certified surgical pathologists. Date of patient death was obtained from the Epidemiological Cancer Registry of the state of Schleswig-Holstein, Germany. Follow-up data of those patients who were still alive were retrieved from hospital records and general practitioners [48]. Of all included patient cases, the pretherapeutic biopsy, primary tumor, lymph node metastasis, and distant metastasis were examined, if available.

### 2.3. Histology

Tissue samples were fixed in formalin and embedded in paraffin. Subsequently, all deparaffinized sections were stained with hematoxylin and eosin (H&E). Histological reexamination of primary tissue sections was carried out for all cases to assure if inclusion criteria were met. Tumors were classified according to the Laurén classification [49] and reexamined by two surgical pathologists. The pTNM stage of all study patients was determined according to the 7th edition of the UICC guidelines [48, 50]. To assess the response to therapy, the amount of tumor residuals with respect to the chemotherapeutic scar was estimated in percentage on the primary tumor sections. Based on this quantitative assessment, all patient cases were divided into two (divided by the median) and four groups (divided into quartiles). In addition, the Becker regression score was determined for each case [51, 52].

### 2.4. Immunhistochemistry

Immunohistochemistry was performed on formalin-fixed and paraffin-embedded sections using antibodies directed against FZD7 (polyclonal, rabbit, Abcam, Cambridge, USA, ab51049, 1 : 200), LGR5 (polyclonal, rabbit, Pineda, Berlin, Germany, not commercial, 1 : 1000), TROY (monoclonal, clone EPR3213(2), rabbit, Abcam, Cambridge, USA, ab138502, 1 : 2000), and MIST1 (monoclonal, clone D7N4B, rabbit, Cell Signaling Technology, Danvers, USA, #14896, 1 : 100). The Leica BOND MAX (Leica Biosystems, Nußloch, Germany) was used for immunostaining of LGR5, TROY, and MIST1 using the BOND polymer refine detection kit (Leica Biosystems, Nußloch, Germany). For MIST1 and TROY immunostaining, deparaffinized tissue sections were pretreated for 20 min with ER2-antigen retrieval solution (Leica Biosystems, Nußloch, Germany).

Immunostaining of FZD7 was done manually. In brief: following antigen retrieval in citrate buffer (125°C), tissue sections were incubated with lab vision hydrogen peroxide block and ultra v block (both Thermo Fisher Scientific GmbH, Schwerte, Germany) in order to avoid unspecific background. Incubation with the primary antibody was done for 30 minutes at room temperature and subsequently overnight at 4°C. Immunoreactions were visualized with the ImmPRESS HRP Universal Antibody and ImmPact NovaRed Peroxidase Substrate (both Vector Laboratories, Burlingame, USA). Between the steps, all slides were washed with Tris-buffered saline (TBS). For counterstaining, hematoxylin (Dr. K. Hollborn & Söhne GmbH & Co. KG, Leipzig, Germany) was used. Immunohistochemical stainings were validated by reverse transcriptase reaction and quantitative real-time PCR (qRT-PCR) on a selected number of cases (Supplementary [Supplementary-material supplementary-material-1]).

### 2.5. Evaluation of Immunostaining

Immunostaining of tumor cells was evaluated according to a modified immunoreactivity scoring system (IRS): Category A documented the maximum intensity of the positive tumor cells as absent (0), weak (1), moderate (2), and strong (3). Category B documented the percentage of positive tumor cells in a marker-specific approach into four grades, i.e., negative (LGR5: 0% positive tumor cells; MIST1: 0%; FZD7: 0%), 1+ (LGR5: 0.1-19% positive tumor cells; MIST1: 0.1-1%; FZD7: 0.1-1%), 2+ (LGR5: 20-49% positive tumor cells; MIST1: 2-10%; FZD7: 2-10%), and 3+ (LGR5: ≥50% positive tumor cells; MIST1: ≥11%, FZD7: ≥11%). This categorization resulted in a more homogeneous distribution of the percentage of stained tumor cells for each individual biomarker. The addition of categories A and B added up to an IRS of 0 to 6. Finally, the median IRS served as a cut-off to differentiate between low/negative and high. In addition to the IRS, we documented the location of positive tumor cells, i.e., tumor surface, tumor center, and invasion front.

### 2.6. Study Design

The study cohort consisted of 119 neoadjuvantly/perioperativley treated patients with GC. Resected primary tumor sites were available in 118 cases, of which 105 still enclosed residuals of the viable primary tumor. In a single patient, the primary tumor could not be analyzed due to technical limitations. Lymph nodes were studied from 79 patients, of which 71 had histological evidence of lymph node metastases. Selection was based on either tumor cells present in the lymph nodes (nodal positive GC) or evidence of tumor regression in lymph nodes, as sometimes, viable tumor cells were found on deeper step sections. 14 distant metastases were available from 10 GC patients, and pretherapeutic biopsies with tumor were available from 25 patients. In total, we studied 236 tissue samples for the presence of LGR5, FZD7, MIST1, and TROY. With regard to primary tumor, the tumor compartments, tumor surface, tumor center, and invasion front were assessed separately. The results were correlated with various clinicopathological patient characteristics and survival.

### 2.7. Statistical Analyses

Statistical analyses were done using SPSS version 24 (IBM, Corp., Aramark, USA). Variables of the ordinal scale were tested with Kendall's tau test and nonordinal variables with Fisher's exact test. Median survival with 95% confidence intervals was determined by the Kaplan-Meier method. Differences between median survivals were tested with the log rank test. Cox regression was used for multivariate analysis. In all tests, a *p* value ≤ 0.05 was defined as statistically significant. The multivariable analysis also included influencing factors with a *p* value < 0.10. The explorative Simes procedure [53] was used for each antibody separately to control the false discovery rate. All *p* values are marked, which remained significant after the Simes procedure.

## 3. Results

The characteristics of our patient cohort are summarized in Table 1. A total of 119 patients fulfilled all study criteria. Survival data were available in 115 (96.6%) cases. The mean follow-up period was 29.5 months (range 0.3 to 117.9 months).

### 3.1. Study Cohort

The study cohort consisted of 119 patients. Primary tumors could be evaluated in 118 cases, pretherapeutic biopsies in 25, lymph node metastases and/or lymph nodes with features of tumor regression in 79, and distant metastases in 14. Thirteen patients (10.9%) showed complete tumor regression of the primary tumor (Becker regression score 1a), of which three still had viable tumor cells in lymph node metastases suitable for histological classification. 25 cases (21%) contained less than 10% of vital tumor residuals in the primary tumor tissue (Becker regression score 1b). 19 (16%) contained 10-50% of vital tumor residuals (Becker regression score 2) and 62 (52.1%) showed more than 50% of vital tumor residuals (Becker regression score 3). 25 patients (21%) had a diffuse, 52 (43.7%) an intestinal, 18 (15.1%) a mixed, and 14 (11.8%) an unclassifiable type of GC according to Laurén [49].

### 3.2. LGR5, FZD7, TROY, and MIST1 Expression in Pretherapeutic Biopsies and Neoadjuvantly Treated Tumor Tissue

#### 3.2.1. LGR5 (Figure 1)

The tumor cells of 88 (87.1%, valid *n* = 101) primary GCs, 54 (77.1%, valid *n* = 70) lymph node metastases, 10 (100%, valid *n* = 10) distant metastases, and 21 (87.5%, valid *n* = 24) pretherapeutic biopsies showed a cytoplasmic expression of LGR5. LGR5 was also found in endothelial cells, nonneoplastic epithelium, stroma cells, myocytes, and fat cells as described by Simon et al. [16].

In the primary tumor, LGR5 was found in the tumor center in 72 (71.3%; valid *n* = 101) and at the invasion front in 71 (70.3%) primary GCs. The tumor surface could only be evaluated in 81 cases, of which 57 (70.4%) expressed LGR5. While the tumor center and invasion front correlated with each other (*p* < 0.001), there was no correlation between the tumor surface and tumor center (*p* = 0.061) or tumor surface and invasion front (*p* = 0.032, not significant after multiple testing; data not shown).

#### 3.2.2. MIST1 (Figure 2)

Nuclear immunostaining of MIST1 was found in tumor cells of 55 (53.9%; valid *n* = 102) primary GCs, 25 (39.1%; valid *n* = 64) lymph node metastasis, 4 (44.4%; valid *n* = 9) distant metastases, and 6 (26.1%; valid *n* = 23) pretherapeutic biopsies.

Tumor cells expressed MIST1 in the tumor center of 51 (50.0%, valid *n* = 102) and at the invasion front of 43 (42.2%, valid *n* = 102) GCs. The tumor surface could only be assessed in 80 cases, of which 29 (36.3%) showed MIST1-positive tumor cells. The expression of MIST1 at the tumor surface, tumor center, and invasion front correlated significantly which each other (*p* < 0.001 each; data not shown).

MIST1 was also expressed by inflammatory cells, as previously reported by Lennerz et al. [54], and by epithelial cells of the nonneoplastic mucosa.

#### 3.2.3. FZD7 (Figure 3)

FZD7 was expressed in tumor cells of 73 (73%; valid *n* = 100) primary GCs, 34 (54%; valid *n* = 63) lymph node metastases, 4 (50%; valid *n* = 8) distant metastases, and 10 (45.5%; valid *n* = 22) pretherapeutic biopsies. Cytoplasmic staining was observed in 67 (67%; valid *n* = 100) primary GCs, 34 (54%; valid *n* = 63) lymph node metastases, 4 (50%; valid *n* = 8) distant metastases, and 9 (40.9%; valid *n* = 22) pretherapeutic biopsies. Cell-membrane staining was present in 5 (5%; valid *n* = 100) primary GCs, 1 (1.6%; valid *n* = 63) lymph node metastasis, and 1 (4.5%; valid *n* = 22) pretherapeutic biopsy. Membranous staining was not found in any distant metastasis. In addition, 7 (7%; valid *n* = 100) primary tumors; none of the lymph node metastases, or distant metastases; and 2 (9.1%; valid *n* = 22) pretherapeutic biopsies expressed FZD7 nuclear membrane-bound.

Tumor cells expressed FZD7 in the tumor center of 58 (58%; valid *n* = 100) and at the invasion front of 48 (48%; valid *n* = 100) primary GCs. The tumor surface was assessable in 74 cases, of which 43 (58.1%) expressed FZD7. The expression of FZD7 at the tumor surface, tumor center, and invasion front correlated significantly with each other (surface vs. center: *p* = 0.004; surface vs. invasion front: *p* < 0.001; center vs. invasion front: *p* < 0.001; data not shown).

An expression of FZD7 was also observed in inflammatory cells, endothelial cells, intestinal metaplasia, and cells of the nonneoplastic mucosa.

#### 3.2.4. TROY (Figure 4)

Assessment of TROY immunostaining was cumbersome. Only 1 of 60 valid lymph node metastases (1.7%) and 1 of 14 distant metastases (7.1%), but none of the 100 valid primary tumors and none of the 21 valid pretherapeutic biopsies, expressed TROY in tumor cells. However, TROY was found in myocytes and stroma cells, as already reported by Wilhelm et al. [37]. Nearly all tissue sections, 97% (97 out of 100) of the primary tumor, 90.5% (19 out of 21) of pretherapeutic biopsies, 95% (57 out of 60) of lymph node metastasis, and 100% of distant metastases had a TROY-immunoreactive stroma. The maximum percentage of TROY-positive tumor cells was 5%. Hence, TROY was excluded from further analysis of tumor cells.

### 3.3. Immunoreactivity Score

The distribution patterns of each stem cell marker in the tumor cells of the primary GCs according to the intensity of immunostaining (category A) and with regard to the percentage of immunopositive cells (category B) were summarized (Supplementary [Supplementary-material supplementary-material-1]). For LGR5 and FZD7, the percentage of positive tumor cells varied between 0 and 100%, while the percentage of MIST1-positive tumor cells varied between 0 and 75%. The addition of categories A and B resulted in an IRS ranging from 0 to 6 for each individual case and marker. For statistical analyses, we dichotomized each stem cell marker at the median IRS, i.e. for primary tumor, IRS ≤ 4 vs. ≥5 (LGR5), IRS ≤ 3 vs. ≥4 (FZD7), and IRS = 0 vs. ≥2 (MIST1) (Supplementary [Supplementary-material supplementary-material-1]). Following dichotomization, no significant correlation was found between the expression of any of the markers in the primary GCs (LGR5 vs. MIST1: *p* = 0.687; LGR5 vs. FZD7: *p* = 0.840; and MIST1 vs. FZD7: *p* = 0.310).

### 3.4. Correlation with Therapy Response

Next, we correlated the expression of the different stem cell markers with therapy response according to the Becker regression grade and the percentage of vital tumor cells in the tumor bed.

Interestingly, the expression of LGR5 in the primary GCs correlated significantly with the Becker regression grade as well as with the percentage of vital tumor cells (*p* < 0.001 each; Table 2; Supplementary [Supplementary-material supplementary-material-1]). No correlation was found between the expression of LGR5 in tumor cells of pretherapeutic biopsies and therapy response (Supplementary [Supplementary-material supplementary-material-1]).

The expression of MIST1 in tumor cells of the primary tumor did not correlate with the response to the treatment (Supplementary [Supplementary-material supplementary-material-1]). Interestingly, MIST1 expression in the pretherapeutic biopsy specimens correlated with the Becker regression score (*p* = 0.046) and with the percentage of tumor residuals divided by the median (*p* = 0.048) or divided into quartiles (*p* = 0.012). However, these associations lost significance after multiple testing (Table 2; Supplementary [Supplementary-material supplementary-material-1]).

No correlation was found between FZD7 expression and response to therapy (Supplementary [Supplementary-material supplementary-material-1]).

### 3.5. Correlation with Clinicopathological Patient Characteristics

LGR5 expression in tumor cells of primary GCs correlated only with lymph vessel invasion (L category, *p* = 0.002; Table 1). Also, the expression of LGR5 in lymph node metastases was associated with lymph vessel invasion (*p* = 0.001) (Supplementary [Supplementary-material supplementary-material-1]). No correlation was found between the LGR5 expression in pretherapeutic biopsies and any clinicopathological patient characteristic (Supplementary [Supplementary-material supplementary-material-1]).

MIST1 expression in primary GCs and pretherapeutic biopsies did not correlate with any clinicopathological patient characteristic (Supplementary [Supplementary-material supplementary-material-1]). To the contrary, MIST1 expression in lymph node metastases correlated significantly with the number of lymph node metastases (*p* = 0.004): a strong expression of MIST1 in lymph node metastases was associated with a higher N category (Table 1).

FZD7 expression did not correlate with any of the characteristics, either in the primary tumors or in the pretherapeutic biopsies or in lymph node metastases (Supplementary [Supplementary-material supplementary-material-1]).

### 3.6. Correlation between the Expression in Primary Tumors and the Expression in Lymph Node Metastases/Distant Metastases

The expression of the three stem cell markers in primary GCs revealed a significant association with their expression in lymph node metastasis (LGR5: *p* = 0.001; MIST1: *p* = 0.002; and FZD7: *p* = 0.004; data not shown). However, there was also a large group of discordant cases that did not match the score of primary tumors and the lymph node metastasis. In the small number of evaluable distant metastases, no marker showed a significant correlation between the expression in the primary GCs and the expression in distant metastasis (LGR5: *p* = 0.133; MIST1: *p* = 0.167; and FZD7: *p* = 0.467; data not shown).

### 3.7. Comparison of Pretherapeutic and Posttherapeutic Expression

There was no association between the expression in pretherapeutic biopsies and the expression in neoadjuvant/perioperative treated primary tumors for any marker (LGR5: *p* = 0.087; MIST1: *p* = 0.642; and FZD7: *p* = 0.637; Supplementary [Supplementary-material supplementary-material-1]).

### 3.8. Prognostic Significance

**Figure 5:**
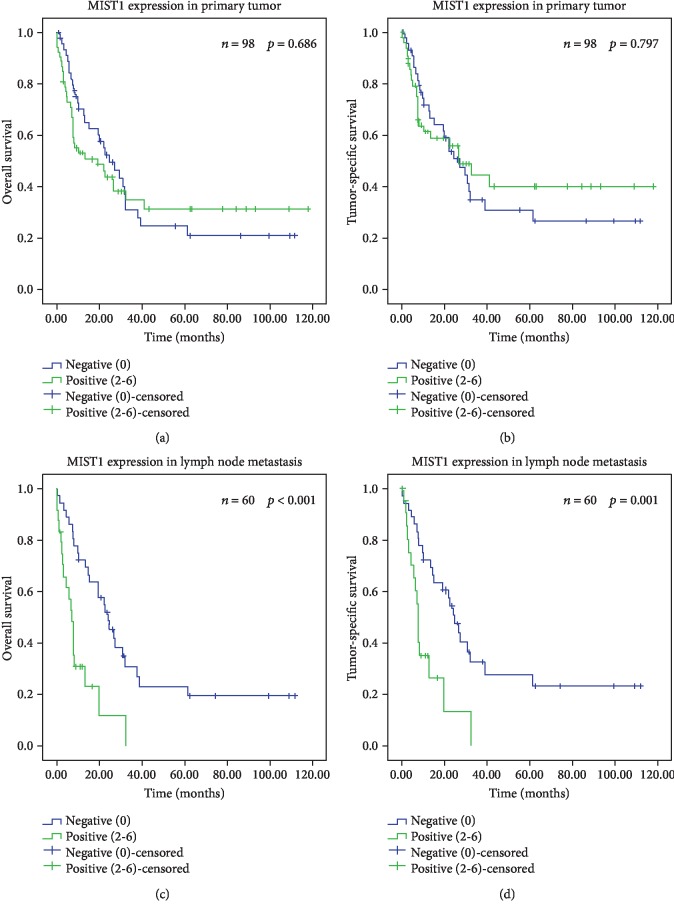
Survival analysis of MIST1 using Kaplan-Meier plots. Kaplan-Meier curves depicting overall survival of the validation cohort according to the MIST1 expression in the primary tumor (a) and lymph node metastasis (c) as well as tumor-specific survival of the validation cohort according to the MIST1 expression in the primary tumor (b) and lymph node metastasis (d).

Patient prognosis significantly depended on several clinicopathological parameters (Supplementary [Supplementary-material supplementary-material-1]) as well as on the MIST1 expression in tumor cells of the lymph node metastasis (Figure 5; Table 1). Patients with MIST1 expression in metastatic tumor cells showed significantly worse overall (*p* < 0.001) and tumor-specific survival (*p* = 0.001).

For LGR5, no relationship with patient survival was found (Table 1, Supplementary [Supplementary-material supplementary-material-1]). The survival analysis was also analyzed separately for each histoanatomical location, i.e., tumor surface, tumor center, and invasion front. However, LGR5 expression did not correlate with patient survival (data not shown). Patient prognosis also did not correlate with FZD7 (Supplementary [Supplementary-material supplementary-material-1]).

## 4. Discussion

The cancer stem cell model is considered as a putative cause of resistance to chemotherapy and disease recurrence in diverse malignant tumors, including GC. While the CSC model is now generally accepted, classification of single biomarkers such as CSC markers is cumbersome and often based on observations made in model systems, i.e., mouse or cell culture experiments. In addition, proof of their significance in clinical samples is scarce or even lacking. In our study, we aimed to fill this gap of information and studied the expression and tumor biological significance of four gastric CSC markers in a cohort of 119 patients with neoadjuvantly/perioperativley treated GCs.

Our selection of four CSC markers for GC, i.e., LGR5, FZD7, MIST1, and TROY, was based on either cell culture experiments using side populations (as surrogate for CSC), lineage tracing experiments in mice, or studies on clinical samples providing evidence of a stem cell marker [13–16, 25, 26, 28, 30, 31, 37, 40–42, 46, 47, 55, 56].

All four markers were detected in our cohort, albeit with variable prevalence and histoanatomical distributions. We observed a coexpression of LGR5, FZD7, and MIST1 in 41.2% of GCs and a complete lack only in 6.2% of the cases. The expression was not interrelated (i.e., between LGR5, FZD7, and MIST1) and may reflect the coexistence of different CSC phenotypes supporting the contention that cancers can harbor heterogeneous and biologically distinct populations of CSCs [57]. Wang et al. provided evidence for the metastatic potential of LGR5 cells: knockdown of LGR5 arrested tumor cell proliferation and invasion [58]. FZD7, in turn, is the predominant Wnt receptor responsible for transmitting Wnt signaling in gastric tumor cells and plays an essential role in tumorigenesis [29]. Interestingly, we could show that the expression of LGR5, FZD7, and MIST1 in the primary tumors correlated significantly with their expression in the corresponding lymph node metastases. These findings lead to the conjecture that LGR5-, FZD7-, and MIST1-positive tumor cells have metastatic potential, in line with observations made in mouse models [29, 58].

With regard to LGR5- and FZD7-positive GCs, their prevalence was higher in neoadjuvantly/perioperatively treated primary tumors compared with treatment-naïve GCs, i.e., 87% vs. 50% for LGR5 [16, 17, 59] and 73% vs. 34% for FZD7 [28]. Our findings are in line with those of Xi et al., who studied the expression of LGR5 in neoadjuvantly treated GCs of a Chinese patient collective. Their number of LGR5-positive GCs reached 66% and was significantly higher compared with treatment-naïve tumors [20]. Collectively, these data support the notion that neoadjuvant/perioperative chemotherapy of GC leads to proportional increase of tumor cells expressing stem cell markers, which cannot be predicted by an analysis of pretherapeutic biopsies.

The increased expression of LGR5 correlated significantly with tumor regression. While tumor regression decreases the overall tumor mass, our IRS documented percentage of tumor cells present and the intensity of immunostaining, both being independent from the total tumor mass present in a given specimen. Thus, we believe that our data support the CSC model at least for LGR5-positive tumors: neoadjuvant/perioperative chemotherapy augmented the expression of stem cell markers. To the contrary, we were unable to detect a significant correlation (after multiple testing) between tumor regression and the expression of MIST1 or FZD7, in both pretherapeutic biopsies and resected primary GCs. This could be related to the cohort size at least for MIST1, which showed an insignificant (after multiple testing) correlation with Becker regression score in pretherapeutic tumor samples. Alternatively, not all stem cell markers may respond to neoadjuvant/perioperative chemotherapy in a similar way.

This is further exemplified when histoanatomical distribution is considered. While the expression of MIST and FZD7 at the tumor surface, tumor center, and invasion front was interrelated significantly, no such interrelation between different tumor compartments was found for LGR5. In addition, the expression of MIST1 in lymph node metastases correlated significantly with the number of lymph node metastases as well as overall and tumor-specific survival. No such correlation was found for MIST-positive tumor cells in the primary GC. Collectively, these data illustrate the complexity of CSC biology with regard to spatial distribution, response to therapy, and prognostic significance. Different anatomical compartments/microenvironments (e.g., primary tumor and metastatic site) may reflect different niches necessitating distinct stem cell characteristics.

Our results also point towards therapeutic potentials. The combination of targeted elimination of LGR5 expressing cells and chemotherapy could improve therapeutic efficacy. In addition, inhibition of MIST1 before the onset of chemotherapy might improve response rates. Neoadjuvant treatment leads to an increased prevalence of FZD7 expression in GC making it an attractive therapeutic target after “induction” chemotherapy: Flanagan et al. could already show the therapeutic potential for FZD7, and further studies on this topic are warranted [29].

Only few tumor cells expressed TROY in our cohort. We used the same antibody as Wilhelm et al. [37], who detected TROY-positive tumor cells in 51% of their treatment-naïve GCs. Thus, neoadjuvant/perioperative treatment seems to reduce the expression of TROY in tumor cells. The stroma retained the expression in nearly all cases. Wilhelm et al. had shown that TROY is significantly more commonly expressed in intestinal compared with diffuse type GC and correlates inversely with the tumor grade and the nodal spread. In the intestinal type, loss of TROY expression was also associated with a significantly worse overall survival [37]. Our findings indicate that neoadjuvant/perioperative treatment of GC is not able to restore the expression of TROY and even further reduces its expression.

Summing up, our study on clinical patient samples shows that (1) GCs may coexpress independently different stem cell markers; (2) neoadjuvant/perioperative treatment of GC significantly impacts on the expression of these different stem cell markers, (3) which cannot be predicted by the analysis of pretherapeutic biopsies; and (4) their expression and tumor biological effect are heterogeneous and have to be viewed as a function of histoanatomical distribution, i.e., microenvironmental cues.

## Figures and Tables

**Figure 1:**
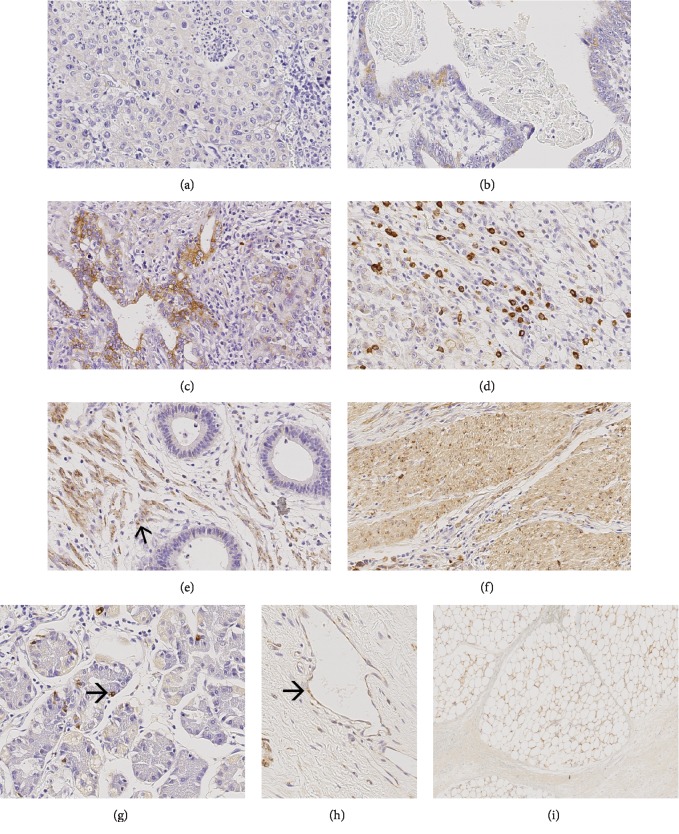
LGR5 expression in neoadjuvant-/perioperative-treated primary tumors. Ascending intensity of LGR5 expression in tumor cells (a–d). LGR5 is also expressed in desmoplastic stroma (e), in myocytes (f), in healthy mucosa cells (g), in endothelial cells (h), and in fat cell membranes (i). Original magnifications: 400-fold.

**Figure 2:**
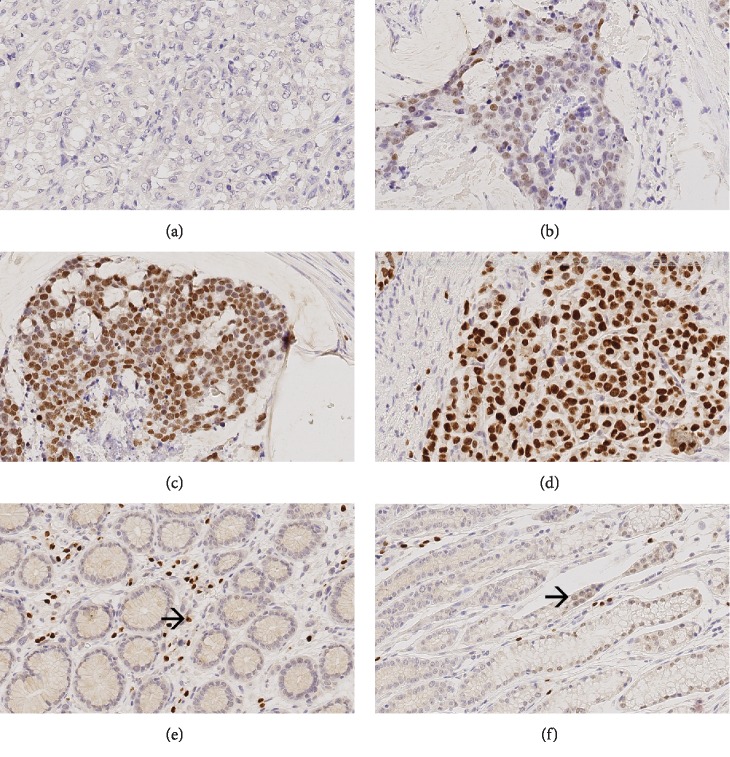
MIST1 expression in neoadjuvant-/perioperative-treated primary tumors. Ascending intensity of MIST1 expression in tumor cells (a–d). MIST1 is also expressed in inflammatory cells (e) and in cells of the healthy mucosa (f). Original magnifications: 400-fold.

**Figure 3:**
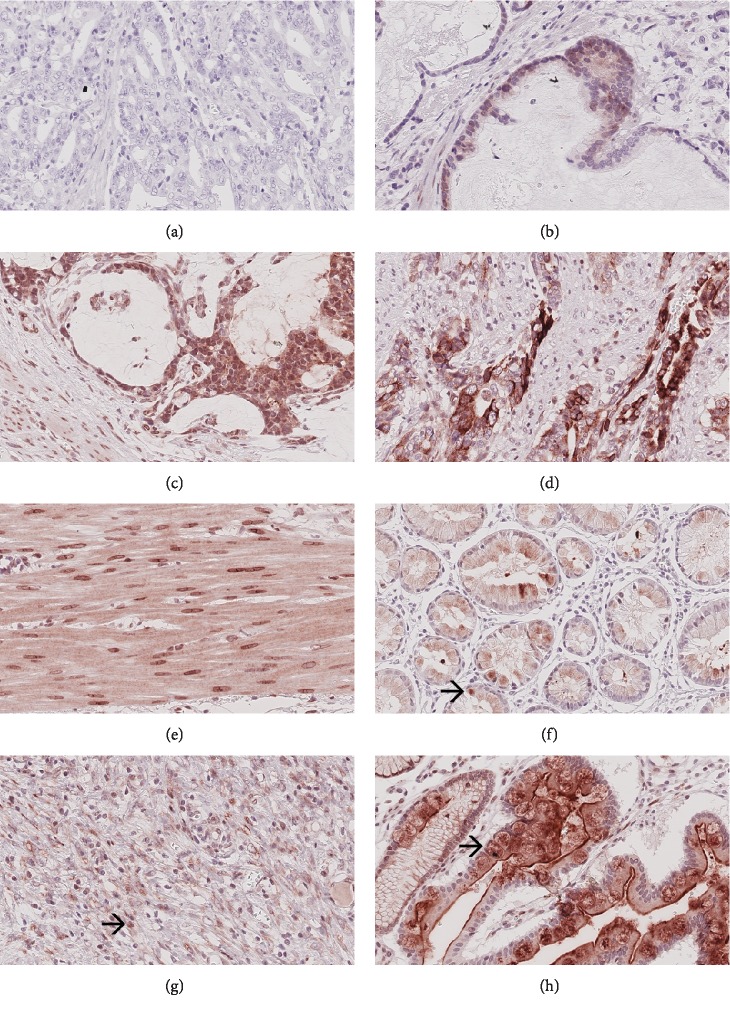
FZD7 expression in neoadjuvant-/perioperative-treated primary tumors. Ascending intensity of FZD7 expression in tumor cells (a–d). FZD7 is also expressed in myocytes (e), in cells of the healthy mucosa (f), in inflammatory cells (g), and in metaplastic cells (h). Original magnifications: 400-fold.

**Figure 4:**
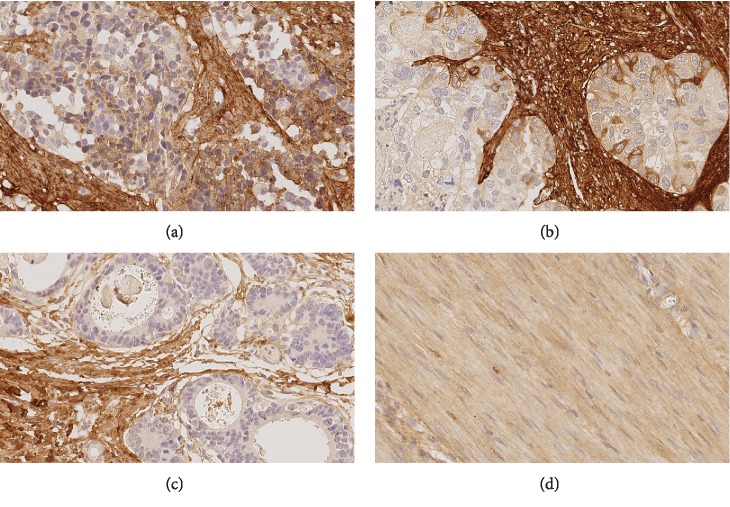
TROY expression in neoadjuvant-/perioperative-treated primary tumors. Expression of TROY in tumor cells of a lymph node (a) and a distant metastasis (b). TROY is also expressed in desmoplastic stromal cells (c) and in myocytes (d). Original magnifications: 400-fold.

**(a):** 

			Valid		Primary resected tumor				Lymph node metastasis			
					LGR5 score 0-6				MIST1 score 0-6			
					Low (0-4)		High (5-6)		Negative (0)		Positive (2-6)	
			*n*	(%)	*n*	(%)	*n*	(%)	*n*	(%)	*n*	(%)
Gender	*n*	*p* ^(1)^			101			0.797	64			0.728
Male			95	(79.8)	47	(56.6)	36	(43.4)	32	(59.3)	22	(40.7)
Female			24	(20.2)	11	(61.1)	7	(38.9)	7	(70.0)	3	(30.0)
Age	*n*	*p* ^(1)^			101			0.423	64			0.204
<64 years			60	(50.4)	31	(62.0)	19	(38.0)	24	(68.6)	11	(31.4)
≥64 years			59	(49.6)	27	(52.9)	24	(47.1)	15	(51.7)	14	(48.3)
Laurén	*n*	*p* ^(1)^			101			0.100	64			0.951
Intestinal			52	(43.7)	30	(61.2)	19	(38.8)	17	(63.0)	10	(37.0)
Diffuse			25	(21.0)	17	(70.8)	7	(29.2)	9	(64.3)	5	(35.7)
Mixed			18	(15.1)	8	(44.4)	10	(55.6)	8	(57.1)	6	(42.9)
Unclassifiable			14	(11.8)	3	(30.0)	7	(70.0)	5	(55.6)	4	(44.4)
Complete regression			10	(8.4)								
Localization	*n*	*p* ^(1)^			101			0.099	64			0.188
Proximal			74	(62.2)	32	(50.8)	31	(49.2)	23	(54.8)	19	(45.2)
Distal			45	(37.8)	26	(68.4)	12	(31.6)	16	(72.7)	6	(27.3)
ypT category	*n*	*p* ^(2)^			101			0.020	64			0.029
ypT0			13	(10.9)					3	(100.0)	0	(0.0)
ypT1a/T1b			19	(16.0)	13	(76.5)	4	(23.5)	1	(50.0)	1	(50.0)
ypT2			15	(12.6)	10	(66.7)	5	(33.3)	9	(81.8)	2	(18.2)
ypT3			63	(52.9)	32	(53.3)	28	(46.7)	24	(57.1)	18	(42.9)
ypT4a/T4b			9	(7.6)	3	(33.3)	6	(66.7)	2	(33.3)	4	(66.7)
ypN category	*n*	*p* ^(2)^			101			0.015	64			0.004^#^
ypN0			44	(37.0)	22	(71.0)	9	(29.0)				
ypN1			27	(22.7)	17	(68.0)	8	(32.0)	18	(81.8)	4	(18.2)
ypN2			25	(21.0)	9	(39.1)	14	(60.9)	13	(61.9)	8	(38.1)
ypN3			23	(19.3)	10	(45.5)	12	(54.5)	8	(38.1)	13	(61.9)
M category	*n*	*p* ^(1)^			101			0.797	64			1.000
M0			98	(82.4)	47	(56.6)	36	(43.4)	32	(61.5)	20	(38.5)
M1			21	(17.6)	11	(61.1)	7	(38.9)	7	(58.3)	5	(41.7)
UICC stage	*n*	*p* ^(2)^			101			0.283	64			0.099
0/0/N+			12	(10.1)					2	(100.0)	0	(0.0)
IA/IB			19	(16.0)	13	(72.2)	5	(27.8)	0	(0)	0	(0)
IIA/IIB			21	(17.6)	13	(61.9)	8	(38.1)	9	(90.0)	1	(10.0)
IIIA/IIIB/IIIC			46	(38.7)	21	(47.7)	23	(52.3)	21	(52.5)	19	(47.5)
IV			21	(17.6)	11	(61.1)	7	(38.9)	7	(58.3)	5	(41.7)

**(b):** 

			Valid		Primary resected tumor				Lymph node metastasis			
					LGR5 score 0-6				MIST1 score 0-6			
					Low (0-4)		High (5-6)		Negative (0)		Positive (2-6)	
			*n*	(%)	*n*	(%)	*n*	(%)	*n*	(%)	*n*	(%)
L category	*n*	*p* ^(1)^			101			0.002^#^	64			0.011
L0			84	(70.6)	47	(68.1)	22	(31.9)	27	(75.0)	9	(25.0)
L1			35	(29.4)	11	(34.4)	21	(65.6)	12	(42.9)	16	(57.1)
V category	*n*	*p* ^(1)^			101			0.010	64			0.100
V0			111	(93.3)	57	(61.3)	36	(38.7)	37	(64.9)	20	(35.1)
V1			8	(6.7)	1	(12.5)	7	(87.5)	2	(28.6)	5	(71.4)
Pn category	*n*	*p* ^(1)^			101			0.229	64			0.009
Pn0			95	(79.8)	48	(60.8)	31	(39.2)	33	(71.7)	13	(28.3)
Pn1			24	(20.2)	10	(45.5)	12	(54.5)	6	(33.3)	12	(66.7)
R status	*n*	*p* ^(1)^			101			0.861	64			0.108
R0			106	(89.1)	51	(58.0)	37	(42.0)	35	(66.0)	18	(34.0)
R1			12	(10.1)	6	(50.0)	6	(50.0)	4	(40.0)	6	(60.0)
RX			1	(0.8)	1	(100.0)	0	(0.0)	0	(0.0)	1	(100.0)
Overall survival	*n*	*p* ^(3)^			97			0.679	60			<0.001^#^
Total/events/censored			115/67/48		56/37/19		41/25/16		36/26/10		24/19/5	
Median survival			26.6 ± 2.7		22.4 ± 6.6		22.8 ± 8.6		24.3 ± 3.9		7.0 ± 1.4	
95% C.I.			21.4-31.8		9.5-35.3		6.0-39.6		16.6-32.0		4.3-9.8	
Tumor-specific survival	*n*	*p* ^(3)^			97			0.953	60			0.001^#^
Total/events/censored			115/55/60		56/32/24		41/19/22		36/24/12		24/16/8	
Median survival			29.5 ± 2.8		26.7 ± 4.3		32.0 ± 8.9		24.6 ± 3.0		7.6 ± 0.4	
95% C.I.			24.0-34.9		18.3-35.2		14.5-49.6		18.8-30.4		6.7-8.4	

^(1)^Fisher's exact test; ^(2)^Kendall's tau test; ^(3)^log-rank test; ^#^significant after multiple testing.

**Table 2:** Correlation of LGR5 and MIST1 expression in primary resected gastric cancer and pretherapeutic biopsies, respectively, with tumor regression.

			Valid		Primary resected tumor				Pretherapeutic biopsy			
					LGR5 score 0-6				MIST1 score 0-6			
					Low (0-4)		High (5-6)		Negative (0)		Positive (2-6)	
			*n*	(%)	*n*	(%)	*n*	(%)	*n*	(%)	*n*	(%)
Vital tumor residuals	*n*	*p* ^(1)^			101			<0.001^#^	23			0.048
<Median (0-54%)			59	(49.6)	34	(79.1)	9	(20.9)	9	(100.0)	0	(0.0)
≥Median (55-100%)			60	(50.4)	24	(41.4)	34	(58.6)	8	(57.1)	6	(42.9)
Vital tumor residuals	*n*	*p* ^(2)^			101			<0.001^#^	23			0.012
Quartile 1 (0-4%)			23	(19.3)	8	(100.0)	0	(0.0)	3	(100.0)	0	(0.0)
Quartile 2 (5-54%)			36	(30.3)	26	(74.3)	9	(25.7)	6	(100.0)	0	(0.0)
Quartile 3 (55-89%)			29	(24.4)	14	(50.0)	14	(50.0)	4	(80.0)	1	(20.0)
Quartile 4 (90-100%)			31	(26.1)	10	(33.3)	20	(66.7)	4	(44.4)	5	(55.6)
Becker regression score	*n*	*p* ^(2)^			101			<0.001^#^	23			0.046
1a			13	(10.9)					2	(100.0)	0	(0.0)
1b			25	(21.0)	19	(82.6)	4	(17.4)	2	(100.0)	0	(0.0)
2			19	(16.0)	13	(72.2)	5	(27.8)	5	(100.0)	0	(0.0)
3			62	(52.1)	26	(43.3)	34	(56.7)	8	(57.1)	6	(42.9)

^(1)^Fisher's exact test; ^(2)^Kendall's tau test; ^#^significant after multiple testing.

## Data Availability

The histological and immunohistochemical data used to support the findings of this study are included within the article and particularly also in the supplemental material.

## Supplementary Materials

Supplementary Materials Supplementary File S1: reverse transcriptase reaction and quantitative real-time PCR (qRT-PCR) for validation of the immunohistochemical stainings. Supplementary Figure S1: validation of immunohistochemical staining (IHC) data for the stem cell markers LGR5, TROY, and MIST1. Supplementary Figure S2: intensity groups (0-3) and amount groups (0-3) of positive tumor cells in the primary tumor. The histograms show the distribution patterns of each putative stem cell marker in the tumor cells of the primary gastric carcinomas according to the intensity of immunostaining (category A) and the amount (percentage) of immunopositive cells (category B). LGR5 (a, d); MIST1 (b, e); FZD7 (c, f). Supplementary Figure S3: creation of immunoreactivity score in the primary tumor. The addition of categories A and B (see Figure S1) resulted in an immunoreactivity score ranging from 0 to 6 for each individual case and marker. The immunoreactivity score from 0 to 6 for LGR5 (a), MIST1 (b), and FZD7 (c). Subsequently, the immunoreactivity score was divided by the median for LGR5 (d), MIST1 (e), and FZD7 (f). Supplementary Figure S4: therapy response. The LGR5 expression in primary tumors (dichotomized at the median immunoreactivity score into low and high) correlated significantly with the tumor regression assessed by the percentage of vital tumor cells in the primary tumor (a; *p* < 0.001); MIST1 expression in biopsies (dichotomized at the median immunoreactivity score into low and high) correlated with tumor regression assessed by the percentage of tumor residuals (b; *p* = 0.048). Supplementary Table S5: correlation of LGR5 and MIST1 expression in primary resected gastric cancer and pretherapeutic biopsies, respectively, with tumor regression. Supplementary Table S6: correlation of LGR5, MIST1, and FZD7 expression in pretherapeutic biopsy, primary resected gastric cancer, and lymph node metastases, respectively, with clinicopathological patient characteristics. Immunoreactivity score dichotomized at the median into low and high or negative and positive. Supplementary Figure S7: changes in the immunoreactivity score from pre- to posttherapeutic tissue. This figure illustrates the change of the immunoreactivity score assessed in pretherapeutic biopsy with the expression in the corresponding primary tumor. As shown, there is no systematic up- or downregulation for any of the three putative stem cell markers, i.e., LGR5 (a; *p* = 0.087), MIST1 (b; *p* = 0.642), or FZD7 (c; *p* = 0.637). Supplementary Table S8: correlation of clinicopathological patient characteristics with tumor-specific survival of the patient cohort. Supplementary Figure S9: survival analysis LGR5. Kaplan-Meier curves depicting overall survival of the validation cohort according to the LGR5 expression in primary tumor (a) and lymph node metastasis (c) as well as tumor-specific survival of the validation cohort according to the LGR5 expression in the primary tumor (b) and lymph node metastasis (d). Supplementary Figure S10: survival analysis FZD7. Kaplan-Meier curves depicting overall survival of the validation cohort according to the FZD7 expression in the primary tumor (a) and lymph node metastasis (c) as well as tumor-specific survival of the validation cohort according to the FZD7 expression in the primary tumor (b) and lymph node metastasis (d).

## References

[1] Ferlay J. S. E. M., Soerjomataram I., Ervik M. (2013). GLOBOCAN 2012 v1. 0.. *Cancer Incidence and Mortality Worldwide: IARC CancerBase*.

[2] Kaatsch P., Spix C., Katalinic A., Hentschel S., Luttmann S., Stegmaier C. (2017). *Krebs in Deutschland für 2013/2014*.

[3] Al-Batran S. E., Homann N., Pauligk C. (2017). Effect of neoadjuvant chemotherapy followed by surgical resection on survival in patients with limited metastatic gastric or gastroesophageal junction cancer: the AIO-FLOT3 trial. *JAMA Oncology*.

[4] Cunningham D., Allum W., Stenning S. (2006). Perioperative chemotherapy versus surgery alone for resectable gastroesophageal cancer. *The New England Journal of Medicine*.

[5] Ychou M., Boige V., Pignon J. P. (2011). Perioperative chemotherapy compared with surgery alone for resectable gastroesophageal adenocarcinoma: an FNCLCC and FFCD multicenter phase III trial. *Journal of Clinical Oncology*.

[6] Spoerl S., Novotny A., al-Batran S. E. (2018). Histopathological regression predicts treatment outcome in locally advanced esophagogastric adenocarcinoma. *European Journal of Cancer*.

[7] Prieto-Vila M., Takahashi R. U., Usuba W., Kohama I., Ochiya T. (2017). Drug resistance driven by cancer stem cells and their niche. *International Journal of Molecular Sciences*.

[8] Chen J., Li Y., Yu T.-S. (2012). A restricted cell population propagates glioblastoma growth after chemotherapy. *Nature*.

[9] Chen J., Wang J., Zhang Y. (2014). Observation of ovarian cancer stem cell behavior and investigation of potential mechanisms of drug resistance in three-dimensional cell culture. *Journal of Bioscience and Bioengineering*.

[10] Li X., Lewis M. T., Huang J. (2008). Intrinsic resistance of tumorigenic breast cancer cells to chemotherapy. *Journal of the National Cancer Institute*.

[11] Kumar K. K., Burgess A. W., Gulbis J. M. (2014). Structure and function of LGR5: an enigmatic G-protein coupled receptor marking stem cells. *Protein Science*.

[12] Lau W., Peng W. C., Gros P., Clevers H. (2014). The R-spondin/Lgr5/Rnf43 module: regulator of Wnt signal strength. *Genes & Development*.

[13] Barker N., van Es J. H., Kuipers J. (2007). Identification of stem cells in small intestine and colon by marker gene Lgr5. *Nature*.

[14] Barker N., Huch M., Kujala P. (2010). Lgr5^+ve^ Stem Cells Drive Self-Renewal in the Stomach and Build Long-Lived Gastric Units In Vitro. *Cell Stem Cell*.

[15] Jaks V., Barker N., Kasper M. (2008). Lgr5 marks cycling, yet long-lived, hair follicle stem cells. *Nature Genetics*.

[16] Simon E., Petke D., Böger C. (2012). The spatial distribution of LGR5+ cells correlates with gastric cancer progression. *PLoS One*.

[17] Xi H. Q., Cai A. Z., Wu X. S. (2014). Leucine-rich repeat-containing G-protein-coupled receptor 5 is associated with invasion, metastasis, and could be a potential therapeutic target in human gastric cancer. *British Journal of Cancer*.

[18] Harada Y., Kazama S., Morikawa T. (2017). Leucine-rich repeat-containing G protein-coupled receptor 5 and CD133 expression is associated with tumor progression and resistance to preoperative chemoradiotherapy in low rectal cancer. *Oncology Letters*.

[19] Osawa H., Takahashi H., Nishimura J. (2016). Full-length LGR5-positive cells have chemoresistant characteristics in colorectal cancer. *British Journal of Cancer*.

[20] Xi H.-Q., Cui J.-X., Shen W.-S. (2014). Increased expression of Lgr5 is associated with chemotherapy resistance in human gastric cancer. *Oncology Reports*.

[21] Zhang L., Guo X., Zhang D. (2017). Upregulated miR-132 in Lgr5+gastric cancer stem cell-like cells contributes to cisplatin-resistance via SIRT1/CREB/ABCG2 signaling pathway. *Molecular Carcinogenesis*.

[22] Kemp C. R., Willems E., Wawrzak D., Hendrickx M., Agbor Agbor T., Leyns L. (2007). Expression of Frizzled5, Frizzled7, and Frizzled10 during early mouse development and interactions with canonical Wnt signaling. *Developmental Dynamics*.

[23] Vincan E., Flanagan D. J., Pouliot N., Brabletz T., Spaderna S. (2010). Variable FZD7 expression in colorectal cancers indicates regulation by the tumour microenvironment. *Developmental Dynamics*.

[24] Melchior K., Weiß J., Zaehres H. (2008). the WNT receptor FZD7 contributes to self-renewal signaling of human embryonic stem cells. *Biological Chemistry*.

[25] Flanagan D. J., Barker N., Nowell C. (2017). Loss of the Wnt receptor frizzled 7 in the mouse gastric epithelium is deleterious and triggers rapid repopulation in vivo. *Disease Models & Mechanisms*.

[26] Kirikoshi H., Sekihara H., Katoh M. (2001). Up-regulation of Frizzled-7 (FZD7) in human gastric cancer. *International Journal of Oncology*.

[27] Li G., Su Q., Liu H. (2018). Frizzled7 promotes epithelial-to-mesenchymal transition and stemness via activating canonical Wnt/*β*-catenin pathway in gastric cancer. *International Journal of Biological Sciences*.

[28] Schmuck R., Warneke V., Behrens H.-M., Simon E., Weichert W., Röcken C. (2011). Genotypic and phenotypic characterization of side population of gastric cancer cell lines. *The American Journal of Pathology*.

[29] Flanagan D. J., Barker N., Costanzo N. S. D. (2019). Frizzled-7Is required for Wnt signaling in gastric tumors with and WithoutApcMutations. *Cancer Research*.

[30] Merle P., de la Monte S., Kim M. (2004). Functional consequences of frizzled-7 receptor overexpression in human hepatocellular carcinoma. *Gastroenterology*.

[31] Ueno K., Hazama S., Mitomori S. (2009). Down-regulation of frizzled-7 expression decreases survival, invasion and metastatic capabilities of colon cancer cells. *British Journal of Cancer*.

[32] Yang L., Wu X., Wang Y. (2011). FZD7 has a critical role in cell proliferation in triple negative breast cancer. *Oncogene*.

[33] Chen Z., Ma T., Huang C. (2013). MiR-27a modulates the MDR1/P-glycoprotein expression by inhibiting FZD7/*β*-catenin pathway in hepatocellular carcinoma cells. *Cellular Signalling*.

[34] Liu X., Yan Y., Ma W., Wu S. (2017). Knockdown of frizzled-7 inhibits cell growth and metastasis and promotes chemosensitivity of esophageal squamous cell carcinoma cells by inhibiting Wnt signaling. *Biochemical and Biophysical Research Communications*.

[35] Flanagan D. J., Phesse T. J., Barker N. (2015). Frizzled7 Functions as a Wnt Receptor in Intestinal Epithelial Lgr5^+^ Stem Cells. *Stem Cell Reports*.

[36] Fafilek B., Krausova M., Vojtechova M. (2013). Troy, a tumor necrosis factor receptor family member, interacts with lgr5 to inhibit wnt signaling in intestinal stem cells. *Gastroenterology*.

[37] Wilhelm F., Böger C., Krüger S., Behrens H.-M., Röcken C. (2017). Troy is expressed in human stomach mucosa and a novel putative prognostic marker of intestinal type gastric cancer. *Oncotarget*.

[38] Kojima T., Morikawa Y., Copeland N. G. (2000). TROY, a newly identified member of the tumor necrosis factor receptor superfamily, exhibits a homology with Edar and is expressed in embryonic skin and hair follicles. *The Journal of Biological Chemistry*.

[39] Pispa J., Pummila M., Barker P. A., Thesleff I., Mikkola M. L. (2008). Edar and Troy signalling pathways act redundantly to regulate initiation of hair follicle development. *Human Molecular Genetics*.

[40] Stange D. E., Koo B.-K., Huch M. (2013). Differentiated Troy^+^ chief cells act as reserve stem cells to generate all lineages of the stomach epithelium. *Cell*.

[41] Paulino V. M., Yang Z., Kloss J. (2010). TROY (TNFRSF19) is overexpressed in advanced glial tumors and promotes glioblastoma cell invasion via Pyk2-Rac1 signaling. *Molecular Cancer Research*.

[42] Spanjaard R. A., Whren K. M., Graves C., Bhawan J. (2007). Tumor necrosis factor receptor superfamily member TROY is a novel melanoma biomarker and potential therapeutic target. *International Journal of Cancer*.

[43] Lemercier C., To R. Q., Swanson B. J., Lyons G. E., Konieczny S. F. (1997). Mist1: a novel basic helix-loop-helix transcription factor exhibits a developmentally regulated expression pattern. *Developmental Biology*.

[44] Chikada H., Ito K., Yanagida A., Nakauchi H., Kamiya A. (2015). The basic helix-loop-helix transcription factor, Mist1, induces maturation of mouse fetal hepatoblasts. *Scientific Reports*.

[45] Pin C. L., Rukstalis J. M., Johnson C., Konieczny S. F. (2001). The bHLH transcription factor Mist1 is required to maintain exocrine pancreas cell organization and acinar cell identity. *The Journal of Cell Biology*.

[46] Hayakawa Y., Ariyama H., Stancikova J. (2015). Mist1 expressing gastric stem cells maintain the normal and neoplastic gastric epithelium and are supported by a perivascular stem cell niche. *Cancer Cell*.

[47] Sakitani K., Hayakawa Y., Deng H. (2017). CXCR4-expressing Mist1^+^ progenitors in the gastric antrum contribute to gastric cancer development. *Oncotarget*.

[48] Böger C., Behrens H.-M., Mathiak M., Krüger S., Kalthoff H., Röcken C. (2016). PD-L1 is an independent prognostic predictor in gastric cancer of Western patients. *Oncotarget*.

[49] Laurén P. (1965). The two histological main types of gastric carcinoma: diffuse and so-called intestinal-type CARCINOMA. *Acta Pathologica et Microbiologica Scandinavica*.

[50] Wittekind C., Meyer H.-J. (2010). *TNM: Klassifikation Maligner Tumoren. 7. Auflage*.

[51] Becker K., Mueller J. D., Schulmacher C. (2003). Histomorphology and grading of regression in gastric carcinoma treated with neoadjuvant chemotherapy. *Cancer*.

[52] Becker K., Langer R., Reim D. (2011). Significance of histopathological tumor regression after neoadjuvant chemotherapy in gastric adenocarcinomas: a summary of 480 cases. *Annals of Surgery*.

[53] Benjamini Y., Hochberg Y. (1995). Controlling the false discovery rate: a practical and powerful approach to multiple testing. *Journal of the Royal Statistical Society. Series B (Methodological)*.

[54] Lennerz J. K. M., Kim S.-H., Oates E. L. (2010). The transcription factor MIST1 is a novel human gastric chief cell marker whose expression is lost in metaplasia, dysplasia, and carcinoma. *The American Journal of Pathology*.

[55] Hirsch D., Barker N., McNeil N. (2014). LGR5 positivity defines stem-like cells in colorectal cancer. *Carcinogenesis*.

[56] Yamamoto Y., Sakamoto M., Fujii G. (2003). Overexpression of orphan G‐protein–coupled receptor, Gpr49, in human hepatocellular carcinomas with *β*‐catenin mutations. *Hepatology*.

[57] Visvader J. E., Lindeman G. J. (2012). Cancer stem cells: current status and evolving complexities. *Cell Stem Cell*.

[58] Wang X., Wang X., Liu Y. (2018). LGR5 regulates gastric adenocarcinoma cell proliferation and invasion via activating Wnt signaling pathway. *Oncogenesis*.

[59] Zheng Z.-X., Sun Y., Bu Z.-D. (2013). Intestinal stem cell marker LGR5 expression during gastric carcinogenesis. *World Journal of Gastroenterology*.

