# Supplementary material for: Expression of LGR5, FZD7, TROY, and MIST1 in Perioperatively Treated Gastric Carcinomas and Correlation with Therapy Response
Source: Dis Markers. 2019 Nov 19;2019:8154926. doi: 10.1155/2019/8154926 (PMC6885822; doi:10.1155/2019/8154926)
Supplement: Supplementary Materials — Supplementary File S1: reverse transcriptase reaction and quantitative real-time PCR (qRT-PCR) for validation of the immunohistochemical stainings. Supplementary Figure S1: validation of immunohistochemical staining (IHC) data for the stem cell markers LGR5, TROY, and MIST1. Supplementary Figure S2: intensity groups (0-3) and amount groups (0-3) of positive tumor cells in the primary tumor. The histograms show the distribution patterns of each putative stem cell marker in the tumor cells of the primary gastric carcinomas according to the intensity of immunostaining (category A) and the amount (percentage) of immunopositive cells (category B). LGR5 (a, d); MIST1 (b, e); FZD7 (c, f). Supplementary Figure S3: creation of immunoreactivity score in the primary tumor. The addition of categories A and B (see Figure S1) resulted in an immunoreactivity score ranging from 0 to 6 for each individual case and marker. The immunoreactivity score from 0 to 6 for LGR5 (a), MIST1 (b), and FZD7 (c). Subsequently, the immunoreactivity score was divided by the median for LGR5 (d), MIST1 (e), and FZD7 (f). Supplementary Figure S4: therapy response. The LGR5 expression in primary tumors (dichotomized at the median immunoreactivity score into low and high) correlated significantly with the tumor regression assessed by the percentage of vital tumor cells in the primary tumor (a; p < 0.001); MIST1 expression in biopsies (dichotomized at the median immunoreactivity score into low and high) correlated with tumor regression assessed by the percentage of tumor residuals (b; p = 0.048). Supplementary Table S5: correlation of LGR5 and MIST1 expression in primary resected gastric cancer and pretherapeutic biopsies, respectively, with tumor regression. Supplementary Table S6: correlation of LGR5, MIST1, and FZD7 expression in pretherapeutic biopsy, primary resected gastric cancer, and lymph node metastases, respectively, with clinicopathological patient characteristics. Immunoreactivity score dicho [file 8154926.f1.pdf]

Supplementary File S1

Reverse transcriptase reaction and quantitative real-time PCR (qRT-PCR)

To validate the immunohistochemical stainings, at least four tissue sections not older than 5 years were selected for each marker, where distinct immunohistochemically positive and only weakly or intermediately stained areas were microdissected from 4 - 8 formalin-fixed and paraffin-embedded serial tissue sections. RNA was isolated using RNeasy FFPE Kit (QIAGEN GmbH, Hilden, Germany) and concentration measured using the NanoDrop 2000 device (Thermo Scientific, Darmstadt). For cDNA synthesis, up to 500 ng of total RNA were reverse transcribed with Maxima First Strand cDNA Synthesis Kit (Thermo Fisher Scientific, Schwerte, Germany). Quantitative PCR was carried out using QuantiTect SYBR Green PCR Kit (Qiagen, Hilden, Germany) on a Light Cycler 480 System (Roche, Mannheim, Germany). All assays were performed in duplicates, corrected for efficiency and normalized to succinate dehydrogenase (SDHA) as housekeeping gene by the LightCycler Analysis Software (version 1.5.0 SP3; Roche). The following primer sets were used: SDHA (5’-ATTTGGTGGACAGAGCCTCA-3’ and 5’-CTGGTATCATATCGCAGAGACCT-3’); FZD7 (5’-AAGCGGTTTGGATGAAAAGA-3’ and 5’-GATTCACATCGCCGTTATCA-3’); LGR5 (5’-AATTTGCGAAGCCTTCAATC-3’ and 5’-GGGATTTCTGTTAACGCATTG-3’); TROY (5’-GGAGTGTGTGCCTTGTGGA-3’ and 5’-GCGATCTTCACGAGGTTGA-3’); MIST1 (5’-CCAGCACTACCAGCAGCA-3’ and 5’-AGGACTGGGCGCTAGGTG-3’).

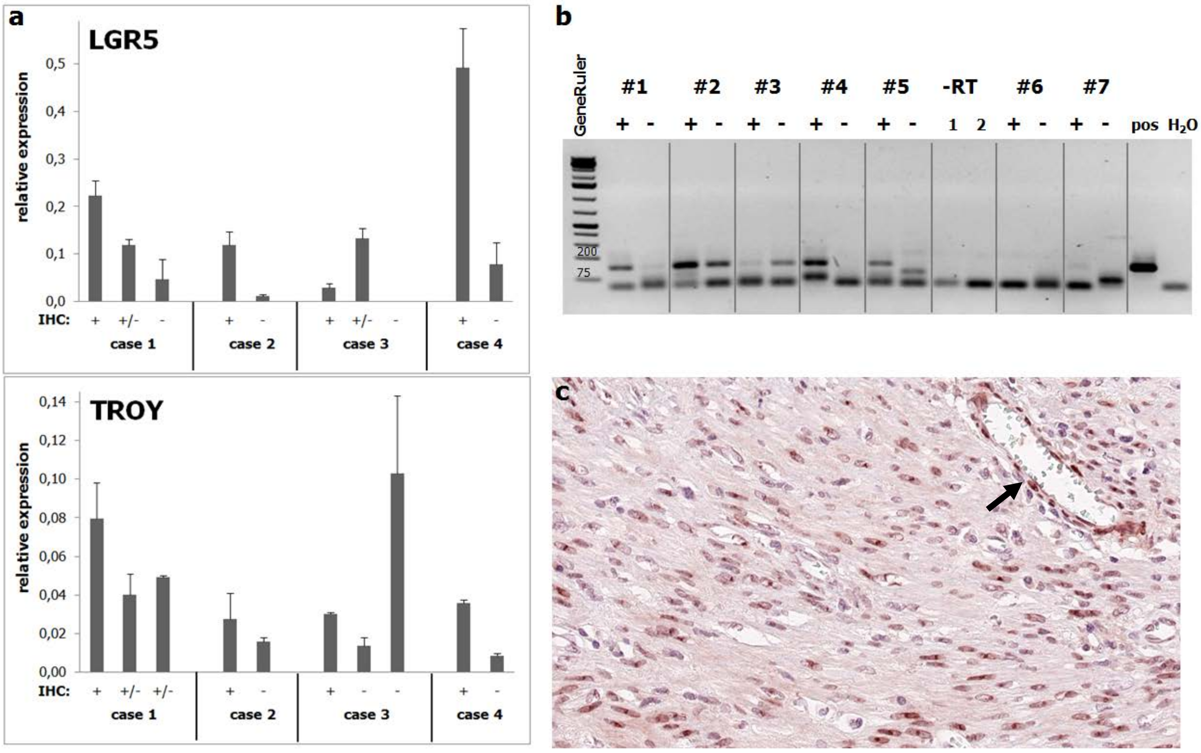

Supplementary Figure S1: Validation of immunohistochemical staining (IHC) data for the stem cell markers LGR5, TROY and MIST1.

A) From microdissected areas, relative expression of LGR5 and TROY was determined by qRT-PCR for four different cases. Values match perfectly to IHC classification with the exception of case3, where the weak cytoplasmic staining of LGR5 in muscle cells (area classified as +/-) results in the detection of large amounts of transcript. Nevertheless, in the dissected area from esophagal tissue (-), the LGR5 transcript is completely absent as expected (paired t-test of the relative expressions between positive and negative areas: p=0.117). For TROY, the second negative area of case 3 shows unexpected high expression levels due to technical problems during preparation and a contamination by strong expressing stromal components (paired t-test of the relative expressions between positive and negative areas: p=0.833).

B) Gel electrophoresis of qRT-PCR products for MIST1. From seven selected cases, enrichment of the specific product at 132 bp was detected for 5 cases in the IHC MIST1^{+} region compared to the IHC MIST1^{-} area. In case 6, no transcript was detected presumably due to low MIST1^{+} cell numbers and low RNA quality. The positive control (reference cDNA, “pos“) shows the specific product, whereas the negative controls (w/o reverse transcriptase “-RT“ and water) only result in primer dimers.

C) Analysis of FZD7 was hampered by high expression in endothelial cells (arrow; anti-FZD7-antibody. Original magnification 400-fold).

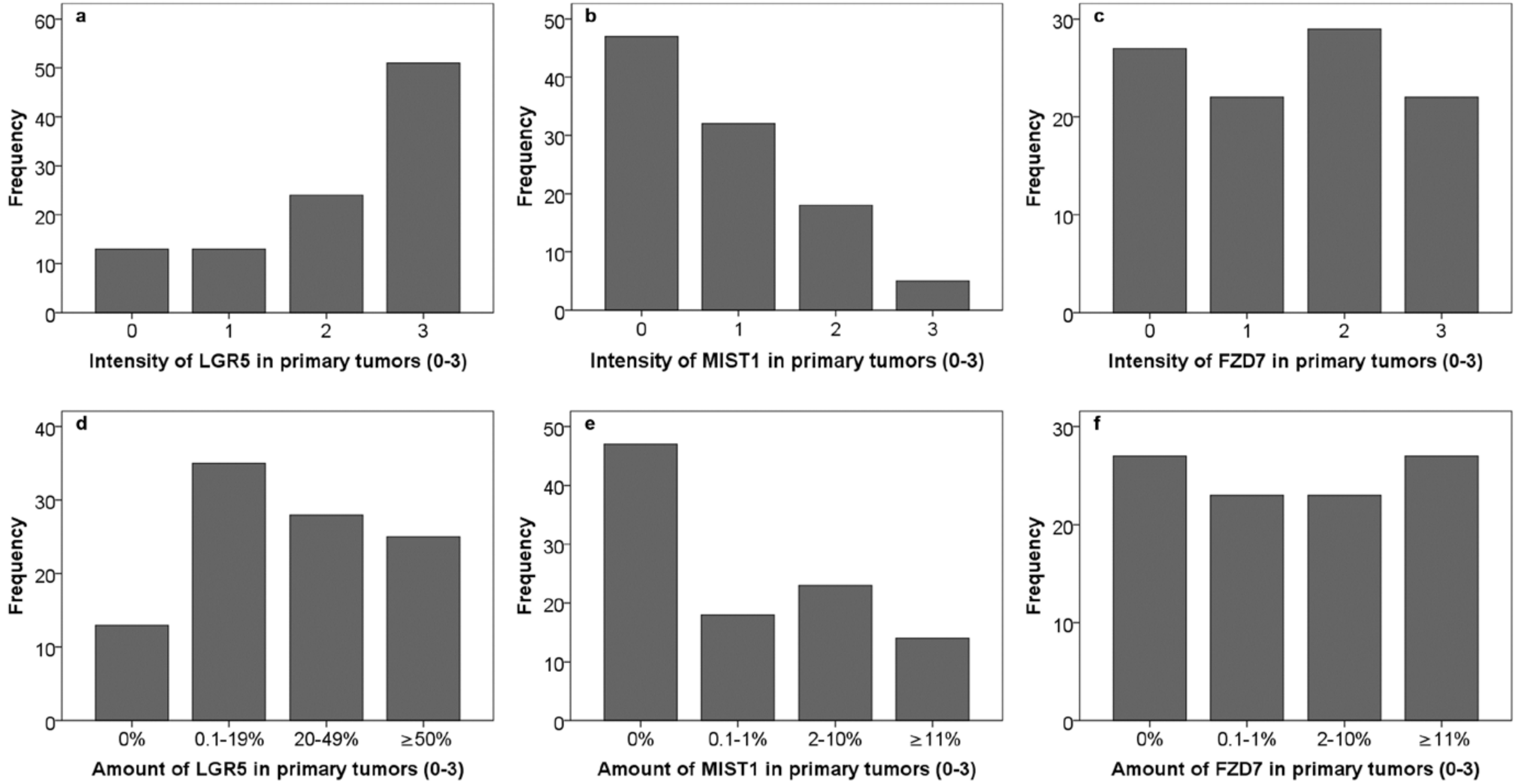

Figure S2 Intensity groups (0-3) and amount groups (0-3) of positive tumor cells in primary tumor.
The histograms show the distribution patterns of each putative stem cell marker in the tumor cells of the primary gastric carcinomas according to the intensity of immunostaining (category A) and the amount (percentage) of immunopositive cells (category B). LGR5 (a, d), MIST1 (b, e), FZD7 (c, f).

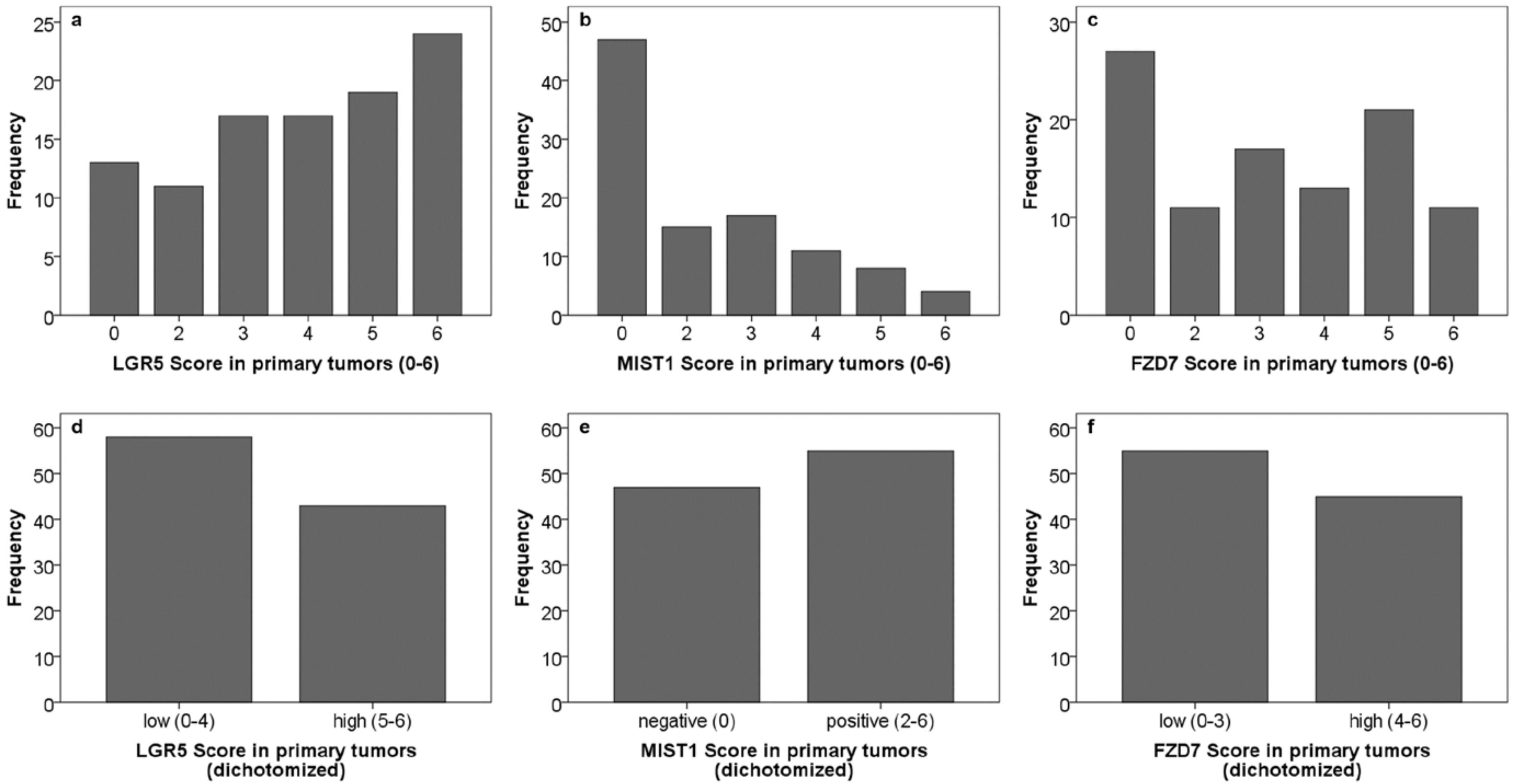

Figure S3 Creation of immunoreactivity score in primary tumor
The addition of category A and B (see Figure S1) resulted in an immunoreactivity score ranging from 0 to 6 for each individual case and marker. Immunoreactivity score from 0 to 6 for LGR5 (a), MIST1 (b), FZD7 (c). Subsequently the immunoreactivity score was divided by the median for LGR5 (d), MIST1 (e), FZD7 (f).

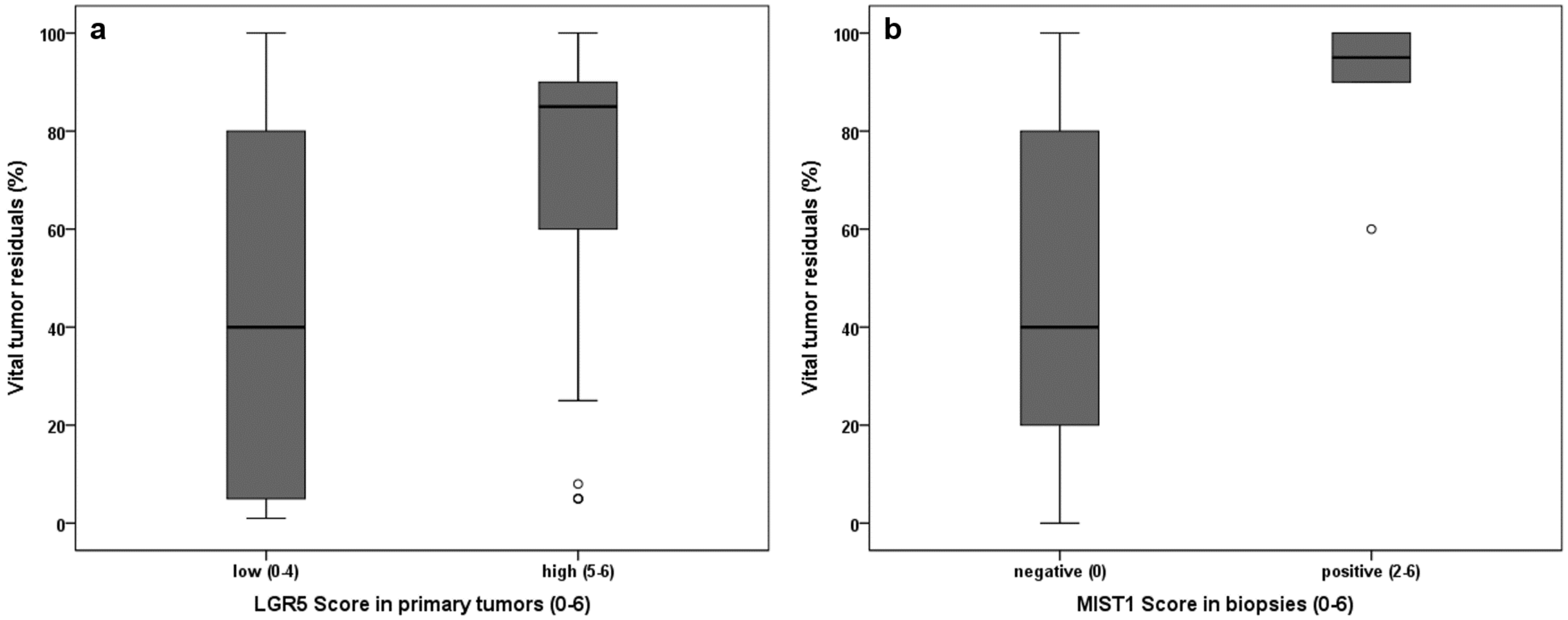

Figure S4 Therapy response
The LGR5 expression in primary tumors (dichotomized at the median immunoreactivity score into low and high) correlated significantly with the tumor regression assessed by the percentage of vital tumor cells in the primary tumor (a; $p<0.001$); MIST1 expression in biopsies (dichotomized at the median immunoreactivity score into low and high) correlated with tumor regression assessed by the percentage of tumor residuals (b; $p=0.048$).

Table S5 Correlation of LGR5- and MIST1-expression in primary resected gastric cancer and pre-therapeutic biopsies, respectively, with tumor regression.

			**Pre-therapeutic biopsy LGR5 Score 0-6**		**Primary resected tumor MIST1 Score 0-6**		**Primary resected tumor FZD7 Score 0-6**		**Pre-therapeutic biopsy FZD7 Score 0-6**	
			**low (0-4)**	**high (5-6)**	**negative (0)**	**positive (2-6)**	**low (0-3)**	**high (4-6)**	**negative (0)**	**positive (2-6)**
			**n (%)**	**n (%)**	**n (%)**	**n (%)**	**n (%)**	**n (%)**	**n (%)**	**n (%)**
**Vital tumor residuals**	**n**	**p**[(1)]	24	0.210	102	0.425	100	0.542	22	1.000
< median (0 - 54%)			2 (22.2)	7 (77.8)	22 (51.2)	21 (48.8)	25 (59.5)	17 (40.5)	5 (55.6)	4 (44.4)
≥ median (55 - 100%)			8 (53.3)	7 (46.7)	25 (42.4)	34 (57.6)	30 (51.7)	28 (48.3)	7 (53.8)	6 (46.2)
**Vital tumor residuals**	**n**	**p**[(2)]	24	0.340	102	0.962	100	0.217	22	0.912
quartile 1 (0 - 4 %)			2 (66.7)	1 (33.3)	5 (55.6)	4 (44.4)	7 (87.5)	1 (12.5)	2 (66.7)	1 (33.3)
quartile 2 (5 - 54 %)			0 (0.0)	6 (100.0)	17 (50.0)	17 (50.0)	18 (52.9)	16 (47.1)	3 (50.0)	3 (50.0)
quartile 3 (55 - 89 %)			3 (50.0)	3 (50.0)	8 (27.6)	21 (72.4)	16 (55.2)	13 (44.8)	2 (40.0)	3 (60.0)
quartile 4 (90 - 100 %)			5 (55.6)	4 (44.4)	17 (56.7)	13 (43.3)	14 (48.3)	15 (51.7)	5 (62.5)	3 (37.5)
**Becker regression score**	**n**	**p**[(2)]	24	0.291	102	0.369	100	0.252	22	1.000
1a			1 (50.0)	1 (50.0)					1 (50.0)	1 (50.0)
1b			1 (50.0)	1 (50.0)	14 (60.9)	9 (39.1)	17 (73.9)	6 (26.1)	1 (50.0)	1 (50.0)
2			0 (0.0)	5 (100.0)	6 (33.3)	12 (66.7)	7 (38.9)	11 (61.1)	3 (60.0)	2 (40.0)
3			8 (53.3)	7 (46.7)	27 (44.3)	34 (55.7)	31 (52.5)	28 (47.5)	7 (53.8)	6 (46.2)

[(1)] Fisher´s Exact Test

[(2)] Kendall's Tau Test

Table S6 Correlation of LGR5-, MIST1- and FZD7-expression in pre-therapeutic biopsy, primary resected gastric cancer and lymph node metastases, respectively, with clinico-pathological patient characteristics. Immunoreactivity score dichotomized at the median into low and high or negative and positive.

			**Pre-therapeutic biopsy LGR5 Score 0-6**		**Lymph node metastasis LGR5 Score 0-6**		**Primary resected tumor MIST1 Score 0-6**	
			**low (0-4)**	**high (5-6)**	**low (0-3)**	**high (4-6)**	**negative (0)**	**positive (2-6)**
			**n (%)**	**n (%)**	**n (%)**	**n (%)**	**n (%)**	**n (%)**
**Gender**	**n**	**p**[(1)]	24	0.239	70	0.381	102	1.000
male			10 (47.6)	11 (52.4)	24 (42.9)	32 (57.1)	39 (46.4)	45 (53.6)
female			0 (0.0)	3 (100.0)	8 (57.1)	6 (42.9)	8 (44.4)	10 (55.6)
**Age**	**n**	**p**[(1)]	24	0.214	70	0.346	102	0.696
< 64 years			7 (58.3)	5 (41.7)	19 (51.4)	18 (48.6)	22 (44.0)	28 (56.0)
≥ 64 years			3 (25.0)	9 (75.0)	13 (39.4)	20 (60.6)	25 (48.1)	27 (51.9)
**Lauren**	**n**	**p**[(1)]	24	0.882	70	0.148	102	0.600
intestinal			4 (50.0)	4 (50.0)	12 (41.4)	17 (58.6)	24 (49.0)	25 (51.0)
diffus			3 (50.0)	3 (50.0)	11 (64.7)	6 (35.3)	13 (52.0)	12 (48.0)
mixed			1 (20.0)	4 (80.0)	7 (50.0)	7 (50.0)	7 (38.9)	11 (61.1)
unclassifiable			1 (33.3)	2 (66.7)	2 (20.0)	8 (80.0)	3 (30.0)	7 (70.0)
complete regression			1 (50.0)	1 (50.0)				
**Localisation**	**n**	**p**[(1)]	24	0.240	70	0.466	102	1.000
proximal			7 (53.8)	6 (46.2)	18 (41.9)	25 (58.1)	29 (45.3)	35 (54.7)
distal			3 (27.3)	8 (72.7)	14 (51.9)	13 (48.1)	18 (47.4)	20 (52.6)
**ypT-category**	**n**	**p**[(2)]	24	0.592	70	0.178	102	0.786
T0			1 (50.0)	1 (50.0)	1 (33.3)	2 (66.7)		
T1a / T1b			1 (50.0)	1 (50.0)	1 (33.3)	2 (66.7)	7 (38.9)	11 (61.1)
T2			1 (33.3)	2 (66.7)	7 (63.6)	4 (36.4)	10 (66.7)	5 (33.3)
T3			7 (46.7)	8 (53.3)	22 (48.9)	23 (51.1)	26 (42.6)	35 (57.4)
T4a / T4b			0 (0.0)	2 (100.0)	1 (12.5)	7 (87.5)	4 (50.0)	4 (50.0)
**ypN-category**	**n**	**p**[(2)]	24	0.665	70	0.105	102	0.983
N0			3 (37.5)	5 (62.5)			13 (40.6)	19 (59.4)
N1			2 (50.0)	2 (50.0)	14 (56.0)	11 (44.0)	13 (54.2)	11 (45.8)
N2			2 (28.6)	5 (71.4)	11 (47.8)	12 (52.2)	13 (54.2)	11 (45.8)
N3			3 (60.0)	2 (40.0)	7 (31.8)	15 (68.2)	8 (36.4)	14 (63.6)
**M-category**	**n**	**p**[(1)]	24	1.000	70	0.381	102	0.613
M0			7 (41.2)	10 (58.8)	24 (42.9)	32 (57.1)	37 (44.6)	46 (55.4)
M1			3 (42.9)	4 (57.1)	8 (57.1)	6 (42.9)	10 (52.6)	9 (47.4)

			Pre-therapeutic biopsy LGR5 Score 0-6		Lymph node metastasis LGR5 Score 0-6		Primary resected tumor MIST1 Score 0-6	
			**low (0-4)**	**high (5-6)**	**low (0-3)**	**high (4-6)**	**negative (0)**	**positive (2-6)**
			**n (%)**	**n (%)**	**n (%)**	**n (%)**	**n (%)**	**n (%)**
**UICC-stage**	**n**	**p**[(2)]	24	1.000	70	0.708	102	0.204
0 / 0/N+			1 (50.0)	1 (50.0)	0 (0.0)	2 (100.0)		
IA / IB			0 (0.0)	1 (100.0)	0 (0)	0 (0)	6 (33.3)	12 (66.7)
IIA / IIB			2 (50.0)	2 (50.0)	7 (63.6)	4 (36.4)	9 (42.9)	12 (57.1)
IIIA / IIIB / IIIC			4 (40.0)	6 (60.0)	17 (39.5)	26 (60.5)	22 (50.0)	22 (50.0)
IV			3 (42.9)	4 (57.1)	8 (57.1)	6 (42.9)	10 (52.6)	9 (47.4)
**L-category**	**n**	**p**[(1)]	24	0.653	70	0.001[#]	102	0.400
L0			8 (47.1)	9 (52.9)	26 (61.9)	16 (38.1)	34 (49.3)	35 (50.7)
L1			2 (28.6)	5 (71.4)	6 (21.4)	22 (78.6)	13 (39.4)	20 (60.6)
**V-category**	**n**	**p**[(1)]	24	1.000	70	0.442	102	0.282
V0			10 (43.5)	13 (56.5)	30 (47.6)	33 (52.4)	45 (47.9)	49 (52.1)
V1			0 (0.0)	1 (100.0)	2 (28.6)	5 (71.4)	2 (25.0)	6 (75.0)
**Pn-category**	**n**	**p**[(1)]	24	0.615	70	0.297	102	0.342
Pn0			9 (45.0)	11 (55.0)	25 (50.0)	25 (50.0)	39 (48.8)	41 (51.2)
Pn1			1 (25.0)	3 (75.0)	7 (35.0)	13 (65.0)	8 (36.4)	14 (63.6)
**R-status**	**n**	**p**[(1)]	24	0.114	70	0.399	102	0.365
R0			10 (50.0)	10 (50.0)	29 (49.2)	30 (50.8)	42 (47.2)	47 (52.8)
R1			0 (0.0)	4 (100.0)	3 (30.0)	7 (70.0)	4 (33.3)	8 (66.7)
RX			0 (0)	0 (0)	0 (0.0)	1 (100.0)	1 (100.0)	0 (0.0)
**Overall survival**	**n**	**p**[(3)]	24	0.218	66	0.649	98	0.686
total/events/censored			10/7/3	14/9/5	30/23/7	36/27/9	46/30/16	52/32/20
median survival			29.5±17.0	13.5±7.0	13.4±4.8	7.8±6.8	24.6±5.3	19.4±9.0
95% C.I.			0.0-62.8	0.0-27.2	3.9-22.9	0.0-21.1	14.2-35.1	1.7-37.1
**Tumorspecific survival**	**n**	**p**[(3)]	24	0.706	66	0.839	98	0.797
total/events/censored			10/6/4	14/6/8	30/20/10	36/22/14	46/27/19	52/24/28
median survival			29.5±15.3	26.7±10.1	15.1±5.6	19.8±10.5	27.3±4.9	26.7±7.0
95% C.I.			0.0-59.4	7.0-46.5	4.1-26.2	0.0-40.3	17.7-37.0	13.1-40.4

Table S6 – continued 1

			Pre-therapeutic biopsy MIST1 Score 0-6				Primary resected tumor FZD7 Score 0-6				Pre-therapeutic biopsy FZD7 Score 0-6			
				negative (0)	positive (2-6)			low (0-3)	high (4-6)			negative (0)	positive (2-6)	
				n (%)	n (%)			n (%)	n (%)			n (%)	n (%)	
**Gender**	**n**	**p**(1)	23			1.000	100			0.434	22			1.000
male				15 (75.0)	5 (25.0)			47 (57.3)	35 (42.7)			10 (52.6)	9 (47.4)	
female				2 (66.7)	1 (33.3)			8 (44.4)	10 (55.6)			2 (66.7)	1 (33.3)	
**Age**	**n**	**p**(1)	23			0.640	100			0.231	22			0.670
< 64 years				8 (66.7)	4 (33.3)			29 (61.7)	18 (38.3)			7 (63.6)	4 (36.4)	
≥ 64 years				9 (81.8)	2 (18.2)			26 (49.1)	27 (50.9)			5 (45.5)	6 (54.5)	
**Lauren**	**n**	**p**(1)	23			0.219	100			0.170	22			0.340
intestinal				5 (62.5)	3 (37.5)			33 (66.0)	17 (34.0)			2 (25.0)	6 (75.0)	
diffus				6 (100.0)	0 (0.0)			10 (41.7)	14 (58.3)			3 (75.0)	1 (25.0)	
mixed				3 (75.0)	1 (25.0)			8 (47.1)	9 (52.9)			4 (80.0)	1 (20.0)	
unclassifiable				1 (33.3)	2 (66.7)			4 (44.4)	5 (55.6)			2 (66.7)	1 (33.3)	
complete regression				2 (100.0)	0 (0.0)							1 (50.0)	1 (50.0)	
**Localisation**	**n**	**p**(1)	23			1.000	100			0.062	22			1.000
proximal				9 (75.0)	3 (25.0)			39 (62.9)	23 (37.1)			7 (53.8)	6 (46.2)	
distal				8 (72.7)	3 (27.3)			16 (42.1)	22 (57.9)			5 (55.6)	4 (44.4)	
**ypT-category**	**n**	**p**(2)	23			0.683	100			0.094	22			0.952
T0				2 (100.0)	0 (0.0)							1 (50.0)	1 (50.0)	
T1a / T1b				2 (100.0)	0 (0.0)			12 (70.6)	5 (29.4)			1 (50.0)	1 (50.0)	
T2				1 (33.3)	2 (66.7)			8 (53.3)	7 (46.7)			2 (66.7)	1 (33.3)	
T3				11 (78.6)	3 (21.4)			33 (55.0)	27 (45.0)			7 (53.8)	6 (46.2)	
T4a / T4b				1 (50.0)	1 (50.0)			2 (25.0)	6 (75.0)			1 (50.0)	1 (50.0)	
**ypN-category**	**n**	**p**(2)	23			0.989	100			0.630	22			0.052
N0				6 (85.7)	1 (14.3)			17 (56.7)	13 (43.3)			3 (37.5)	5 (62.5)	
N1				2 (50.0)	2 (50.0)			15 (60.0)	10 (40.0)			1 (25.0)	3 (75.0)	
N2				5 (71.4)	2 (28.6)			12 (50.0)	12 (50.0)			5 (71.4)	2 (28.6)	
N3				4 (80.0)	1 (20.0)			11 (52.4)	10 (47.6)			3 (100.0)	0 (0.0)	
**M-category**	**n**	**p**(1)	23			1.000	100			0.786	22			1.000
M0				12 (70.6)	5 (29.4)			47 (56.0)	37 (44.0)			9 (52.9)	8 (47.1)	
M1				5 (83.3)	1 (16.7)			8 (50.0)	8 (50.0)			3 (60.0)	2 (40.0)	

			Pre-therapeutic biopsy MIST1 Score 0-6		Primary resected tumor FZD7 Score 0-6		Pre-therapeutic biopsy FZD7 Score 0-6	
			**negative (0)**	**positive (2-6)**	**low (0-3)**	**high (4-6)**	**negative (0)**	**positive (2-6)**
			**n (%)**	**n (%)**	**n (%)**	**n (%)**	**n (%)**	**n (%)**
**UICC-stage**	**n**	**p**[(2)]	23	0.483	100	0.369	22	0.271
0 / 0/N+			2 (100.0)	0 (0.0)			1 (50.0)	1 (50.0)
IA / IB			1 (100.0)	0 (0.0)	14 (77.8)	4 (22.2)	0 (0.0)	1 (100.0)
IIA / IIB			1 (25.0)	3 (75.0)	8 (38.1)	13 (61.9)	1 (25.0)	3 (75.0)
IIIA / IIIB / IIIC			8 (80.0)	2 (20.0)	25 (55.6)	20 (44.4)	7 (70.0)	3 (30.0)
IV			5 (83.3)	1 (16.7)	8 (50.0)	8 (50.0)	3 (60.0)	2 (40.0)
**L-category**	**n**	**p**[(1)]	23	0.632	100	0.832	22	0.162
L0			13 (76.5)	4 (23.5)	38 (55.9)	30 (44.1)	7 (43.8)	9 (56.3)
L1			4 (66.7)	2 (33.3)	17 (53.1)	15 (46.9)	5 (83.3)	1 (16.7)
**V-category**	**n**	**p**[(1)]	23	0.261	100	0.698	22	1.000
V0			17 (77.3)	5 (22.7)	52 (55.9)	41 (44.1)	11 (52.4)	10 (47.6)
V1			0 (0.0)	1 (100.0)	3 (42.9)	4 (57.1)	1 (100.0)	0 (0.0)
**Pn-category**	**n**	**p**[(1)]	23	1.000	100	0.810	22	0.594
Pn0			14 (73.7)	5 (26.3)	44 (55.7)	35 (44.3)	9 (50.0)	9 (50.0)
Pn1			3 (75.0)	1 (25.0)	11 (52.4)	10 (47.6)	3 (75.0)	1 (25.0)
**R-status**	**n**	**p**[(1)]	23	1.000	100	0.864	22	1.000
R0			15 (75.0)	5 (25.0)	48 (53.9)	41 (46.1)	10 (55.6)	8 (44.4)
R1			2 (66.7)	1 (33.3)	6 (60.0)	4 (40.0)	2 (50.0)	2 (50.0)
RX			0 (0)	0 (0)	1 (100.0)	0 (0.0)	0 (0)	0 (0)
**Overall survival**	**n**	**p**[(3)]	23	0.924	96	0.674	22	0.036
total/events/ censored			17/11/6	6/4/2	52/33/19	44/26/18	12/8/4	10/6/4
median survival			29.5±17.3	12.9±2.9	22.4±4.0	24.6±9.1	12.9±2.3	37.8±7.3
95% C.I.			0.0-63.4	7.3-18.6	14.6-30.2	6.8-42.4	8.5-17.4	23.6-52.1
**Tumorspecific survival**	**n**	**p**[(3)]	23	0.933	96	0.200	22	0.017
total/events/ censored			17/8/9	6/3/3	52/30/22	44/18/26	12/7/5	10/3/7
median survival			29.5±20.3	39.0±0.0	22.4±4.1	39.0±9.7	13.5±2.2	39.0
95% C.I.			0.0-69.2		14.4-30.4	20.1-58.0	9.1-17.8	

Table S6 – continued 2

			Lymph node metastasis FZD7 Score 0-6				
			negative (0)		positive (2-6)		
			n	(%)	n	(%)	
**Gender**	**n**	**p**(1)	63				1.000
male			24	(47.1)	27	(52.9)	
female			5	(41.7)	7	(58.3)	
**Age**	**n**	**p**(1)	63				0.614
< 64 years			17	(50.0)	17	(50.0)	
≥ 64 years			12	(41.4)	17	(58.6)	
**Lauren**	**n**	**p**(1)	63				1.000
intestinal			13	(48.1)	14	(51.9)	
diffus			6	(42.9)	8	(57.1)	
mixed			6	(46.2)	7	(53.8)	
unclassifiable			4	(44.4)	5	(55.6)	
complete regression							
**Localisation**	**n**	**p**(1)	63				0.128
proximal			21	(53.8)	18	(46.2)	
distal			8	(33.3)	16	(66.7)	
**ypT-category**	**n**	**p**(2)	63				0.785
T0			1	(50.0)	1	(50.0)	
T1a / T1b			1	(50.0)	1	(50.0)	
T2			4	(36.4)	7	(63.6)	
T3			21	(52.5)	19	(47.5)	
T4a / T4b			2	(25.0)	6	(75.0)	
**ypN-category**	**n**	**p**(2)	63				0.328
N0							
N1			12	(54.5)	10	(45.5)	
N2			9	(42.9)	12	(57.1)	
N3			8	(40.0)	12	(60.0)	
**M-category**	**n**	**p**(1)	63				1.000
M0			24	(45.3)	29	(54.7)	
M1			5	(50.0)	5	(50.0)	

			Lymph node metastasis FZD7 Score 0-6			
			negative (0)		positive (2-6)	
			n	(%)	n	(%)
**UICC-stage**	**n**	**p**(2)	63			0.773
0 / 0/N+			1	(50.0)	1	(50.0)
IA / IB			0	(0)	0	(0)
IIA / IIB			4	(40.0)	6	(60.0)
IIIA / IIIB / IIIC			19	(46.3)	22	(53.7)
IV			5	(50.0)	5	(50.0)
**L-category**	**n**	**p**(1)	63			1.000
L0			17	(47.2)	19	(52.8)
L1			12	(44.4)	15	(55.6)
**V-category**	**n**	**p**(1)	63			0.437
V0			27	(48.2)	29	(51.8)
V1			2	(28.6)	5	(71.4)
**Pn-category**	**n**	**p**(1)	63			0.586
Pn0			19	(43.2)	25	(56.8)
Pn1			10	(52.6)	9	(47.4)
**R-status**	**n**	**p**(1)	63			0.484
R0			23	(43.4)	30	(56.6)
R1			5	(55.6)	4	(44.4)
RX			1	(100.0)	0	(0.0)
**Overall survival**	**n**	**p**(3)	59			0.643
total/events/censored			27/19/8		32/25/7	
median survival			12.9±4.1		13.4±7.5	
95% C.I.			4.9-21.0		0.0-28.2	
**Tumorspecific survival**	**n**	**p**(3)	59			0.256
total/events/censored			27/18/9		32/19/13	
median survival			12.9±5.1		22.1±8.7	
95% C.I.			3.0-22.9		5.1-39.2	

(1) Fisher's Exact Test

(2) Kendall's Tau Test

(3) Log-Rank Test

significant after multiple testing

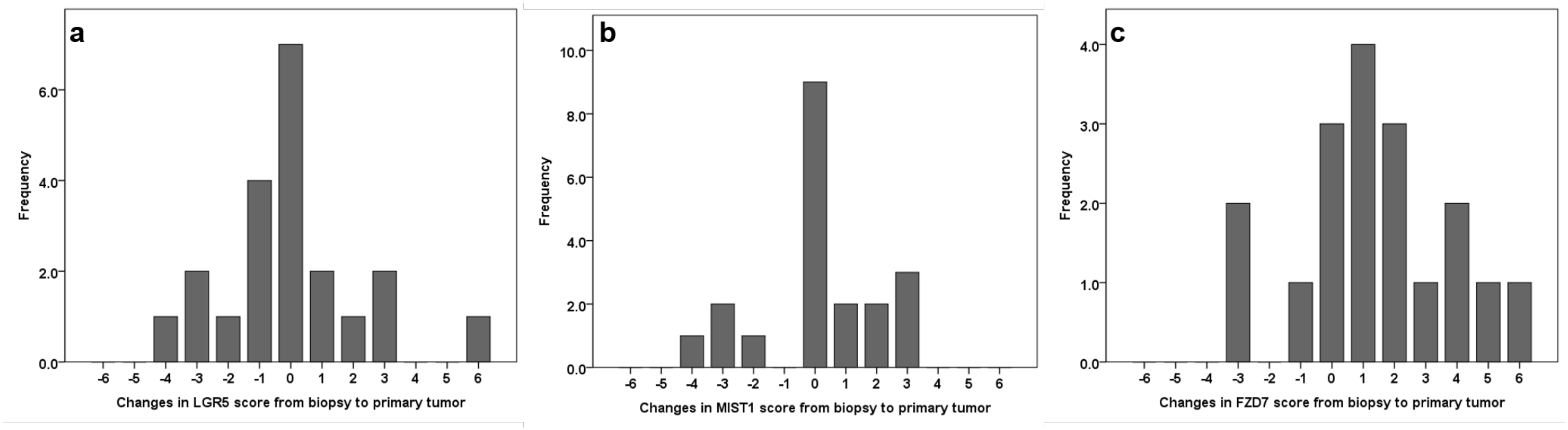

Figure S7 Changes in the immunoreactivity score from pre- to post-therapeutic tissue
This figure illustrates the change of the immunoreactivity score assessed in pre-therapeutic biopsy with the expression in the corresponding primary tumor. As shown, there is no systemic up- or down-regulation for any of the three putative stem cell markes, i.e. LGR5 (a; p= 0.087), MIST1 (b; p=0.642) or FZD7 (c; p=0.637).

Table S8 Correlation of clinico-pathological patient characteristics with tumor-specific survival of the patient cohort

	Patients (n)	Events (n)	Median survival (month; (95% C.I.)	P-value (Logrank-Test)
**Gender**				0.310
male	91	46	27.3 (20.7 - 33.9)	
female	24	9		
**Age**				0.933
< 64 years	58	28	27.3 (10.5 - 44.1)	
≥ 64 years	57	27	30.9 (24.0 - 37.8)	
**Lauren**				0.036
intestinal	51	23		
diffus	24	14		
mixed	16	9		
unclassifiable	14	9		
complete regression	10	0		
**Localisation**				0.284
proximal	71	36	26.7 (20.7 - 32.8)	
distal	44	19	41.0 (0.0 - 86.5)	
**ypT-category**				0.009
T0	13	3		
T1a / T1b	19	4		
T2	14	8	39.0 (0.0 - 100.9)	
T3	60	37	19.8 (8.6 - 31.0)	
T4a / T4b	9	3	13.2	
**ypN-category**				<0.001
N0	44	9		
N1	25	17	24.3 (11.1 - 37.4)	
N2	25	11	22.4 (9.3 - 35.5)	
N3	21	18	7.5 (6.1 - 8.8)	
**M-category**				0.002
M0	95	39	39.0	
M1	20	16	13.5 (1.6 - 25.3)	

	Patients (n)	Events (n)	Median survival (month; (95% C.I.)	P-value (Logrank-Test)
**UICC-stage**				<0.001
0 / 0/N+	12	2		
IA / IB	19	3		
IIA / IIB	19	6		
IIIA / IIIB / IIIC	45	28	13.4 (0.0 - 27.9)	
IV	20	16	13.5 (1.6 - 25.3)	
**L-category**				<0.001
L0	82	30	61.5	
L1	33	25	7.6 (6.6 - 8.7)	
**V-category**				<0.001
V0	107	49	31.7 (20.0 - 43.4)	
V1	8	6	4.9 (3.1 - 6.7)	
**Pn-category**				<0.001
Pn0	93	38	32.3 (2.5 - 62.1)	
Pn1	22	17	7.6 (5.5 - 9.6)	
**R-status**				<0.001
R0	102	45	32.0 (19.5 - 44.5)	
R1	12	9	7.0 (2.3 - 11.8)	
RX	1	1	5.7	
**Vital tumor residuals**				0.021
< median (0 - 54%)	58	25	41.0	
≥ median (55 - 100%)	57	30	22.1 (2.1 - 42.2)	
**Vital tumor residuals**				0.061
quartile 1 (0 - 4 %)	23	10	29.5 (0.0 - 82.4)	
quartile 2 (5 - 54 %)	35	15	41.0	
quartile 3 (55 - 89 %)	27	13	26.7 (13.8 - 39.7)	
quartile 4 (90 - 100 %)	30	17	12.9 (0.5 - 25.4)	
**Becker regression score**				0.054
1a	13	3		
1b	24	12	30.9 (0.0 - 71.9)	
2	19	8	41.0	
3	59	32	22.1 (4.5 - 39.8)	

Grote et al. - Figure S9

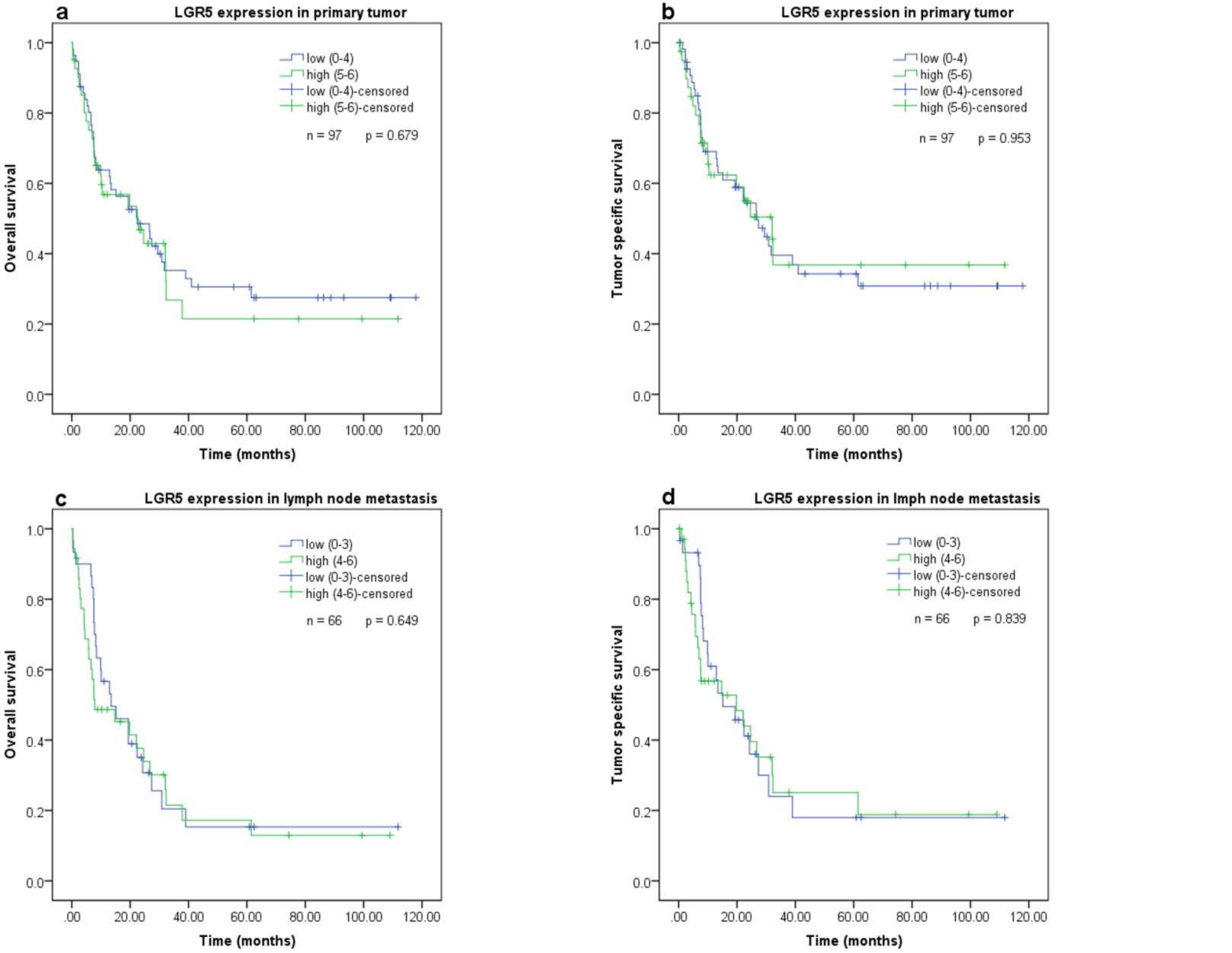

Figure S9 Survival analysis LGR5

Kaplan-Meier curves depicting overall survival of the validation cohort according to the LGR5 expression in primary tumor (a) and lymph node metastasis (c) as well as tumor specific survival of the validation cohort according to the LGR5 expression in primary tumor (b) and lymph node metastasis (d).

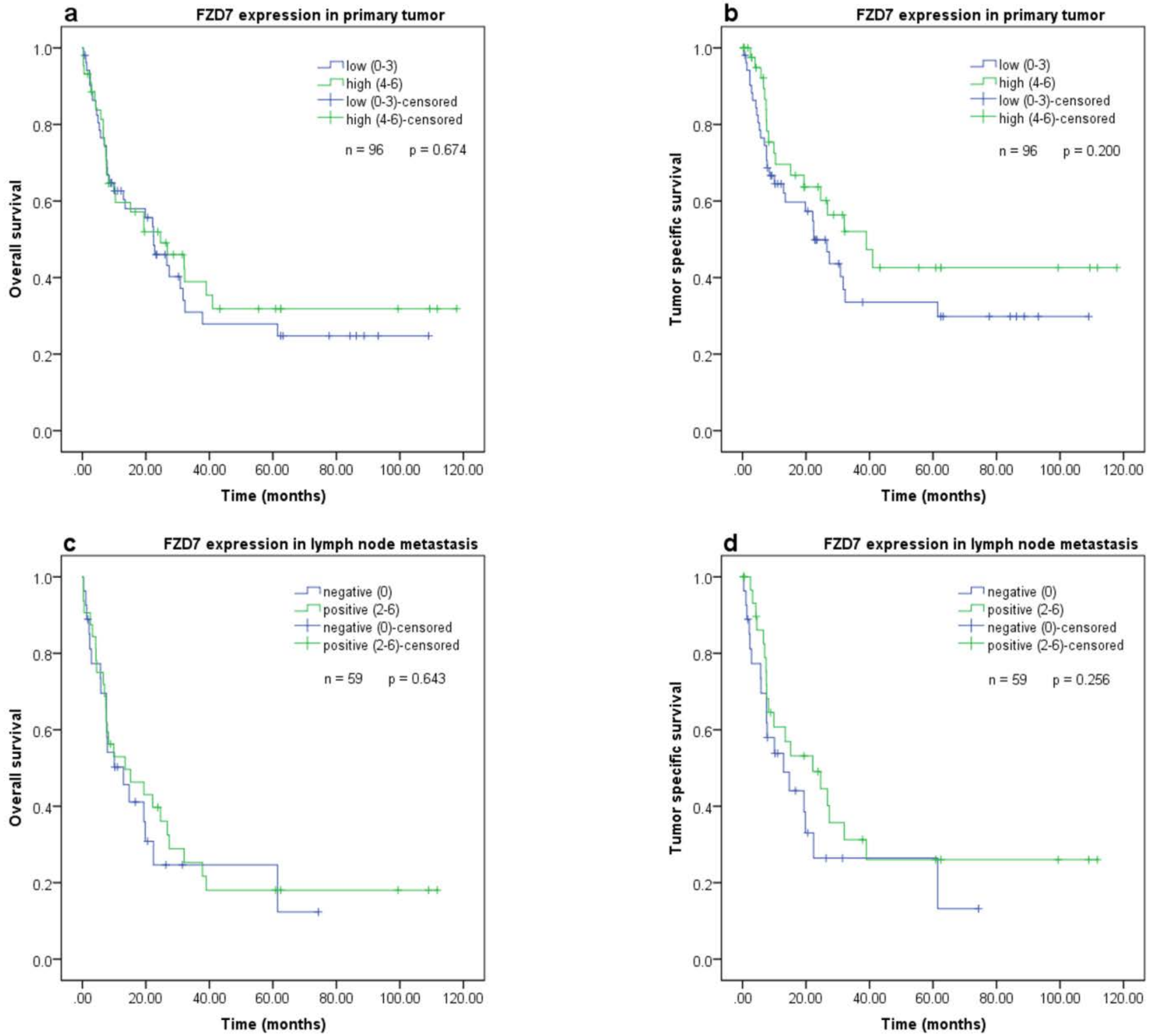

Figure S10 Survival analysis FZD7

Kaplan-Meier curves depicting overall survival of the validation cohort according to the FZD7 expression in primary tumor (a) and lymph node metastasis (c) as well as tumor specific survival of the validation cohort according to the FZD7 expression in primary tumor (b) and lymph node metastasis (d).
